# Intrinsic Structure of Lipoplexes Embedded in Polyelectrolyte Multilayers

**DOI:** 10.1021/acs.langmuir.5c05543

**Published:** 2026-02-13

**Authors:** Maria Krabbes, Vincent Kampik, Mathilde Büttner, Leonard Kaysser, Emanuel Schneck, Chen Shen, Christian Wölk

**Affiliations:** † Institute of Pharmacy, Faculty of Medicine, 9180 Leipzig University, Eilenburger Strasse 15a, 04317 Leipzig, Germany; ‡ Institute for Drug Discovery, Faculty of Medicine, 9180 Leipzig University, Brüderstraße 34, 04103 Leipzig, Germany; § Institute for Condensed Matter Physics, Technische Universität Darmstadt, Hochschulstrasse 8, 64289 Darmstadt, Germany; ∥ Deutsches Elektronen-Synchrotron DESY, Notkestrasse 85, 22607 Hamburg, Germany

## Abstract

The functionalization of surfaces with therapeutically applicable nucleic acid carriers provides promising strategies in biomedical research to develop therapies that focus on local nucleic acid delivery. One such approach is the embedding of lipoplexes (LPXs) in polysaccharide-based polyelectrolyte multilayers (PEMs). PEMs based on hyaluronic acid and chitosan lead to efficient embedding of customized LPX connected with good biological activity. However, although quantitative evaluation demonstrates LPX embedding, information about detailed characteristics of embedded LPXs has been missing. In this study, we used synchrotron-based grazing-incidence small-angle X-ray scattering to investigate the effects of the change in the chemical environment caused by the embedding into PEMs on the LPX’s internal mesoscopic structure. While the lamellar character of the LPXs was preserved, the repeat distance was affected by embedding into polysaccharide-based coatings.

## Graphical abstract

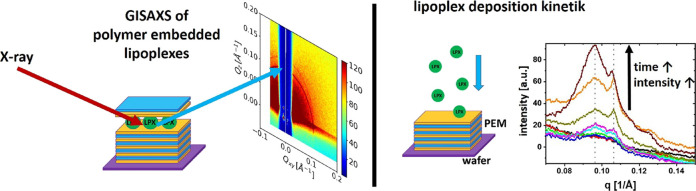

## 1 Introduction

During the past decade, lipid-based nanoparticle systems evolved to an effective class of carriers to deliver therapeutic nucleic acids into cells because of beneficial characteristics regarding e.g. safety, tolerability, ability to redose, transfer capacity, and ability to apply structural design-concepts.^1^ Lipid nanoparticles (LNPs) and lipoplexes (LPXs) are the most prominent lipid-based delivery systems for nucleic acids.

LNPs are lipid-based nanoparticles composed of lipid composites (typically: ionizable lipid, phospholipids, cholesterol, and polyethylene glycol-functionalizes lipid) which complex and encapsulate nucleic acids. The assembling of LNPs base on lipid/nucleic acid complex formation during the direct mixing of an organic lipid solution with an aqueous nucleic acid solution.^2,3^ LNPs are soft-matter nanoparticles which can be characterized by a liquid crystalline inner structure e.g., lamellar or inverse hexagonal.^2^

LPX, too, are lipid-based nanoparticles composed of lipid composites (usually a cationic or ionizable lipid combined with phospholipids) complexing nucleic acids, while here the nucleic acid is complexed with preformulated cationic liposomes.^4^ Various internal liquid crystalline structures have been reported for LPX; including lamellar and (inverse) hexagonal mesophases as well as different types of cubic structures.^4–9^ Although many investigations were aimed at correlating the mesophase structure with LPX efficacy, no general correlations have been established so far.

Commonly, the LNPs and LPX can be applied systemically via i.v. injection or locally (e.g., in muscular tissue for vaccination).^1^ Hence, biomedical applications of nucleic acid therapeutics can benefit from nanoparticle reservoirs for local release.

A promising strategy to implement nanoparticle reservoirs is the application of hydrogels or solid scaffolds loaded with nucleic acid carriers as gene-activated matrices in the field of regenerative medicine for e.g., cartilage or wound regeneration.^10,11^ Special cases are gene-activated surface coatings based on polyelectrolyte multilayers (PEMs) with embedded LPXs, a strategy aimed at realizing substrate-mediated gene delivery. Current LPX-loaded PEMs have different designs but are composed of at least one polysaccharide component: Liu et al. designed a PEM coating based on hyaluronic acid and LPXs;^12^ Holmes and Tabrizian proposed a coating strategy based on hyaluronic acid, chitosan, and LPX;^13^ a minimalistic coating of polyallylamine, chondroitin sulfate, poly-l-lysine, and LPX was designed by Carvalho et al.^14^ Husteden et al. investigated two different systems embedding LPXs, either in chondroitin sulfate/collagen PEMs,^15^ or in hyaluronic acid/chitosan PEMs.^16^ Although the binding and embedding of LPXs in the PEM systems was proven and also quantification approaches were presented, the effect of the embedding process on the internal structure of the LPXs was never studied.

In the present work, we investigate the effect of the interaction of LPX with the polyelectrolytes of the PEMs on the mesoscopic LPX structure. The key technique was grazing incidence small-angle X-ray scattering (GISAXS), a technique that allowed us to determine the LPX mesostructure on ultrathin films like PEM coatings. As test system, PEMs composed of hyaluronic acid (HA) and chitosan (CHI) were chosen (see Figure 1), because the preparation conditions for these materials were intensively studied in our group.^16,17^ Further, the embedded LPX composite, composed of the ionizable lipid OH4 (designed in our group) and the colipid DOPE (structures of both lipids are given in Figure 1), has already been physicochemically characterized as nanoparticles in dispersion.^18^ Our findings demonstrate that the embedding of LPXs in the PEMs can affect their inner mesoscopic structure.

**Figure 1:**
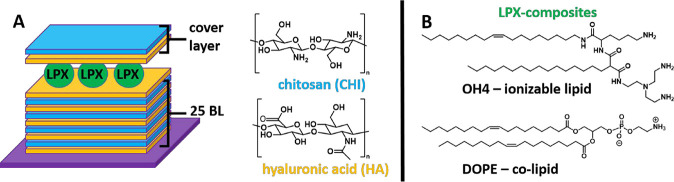
(A) Schematic illustration of the LPX-functionalized PEM system. The PEM components are chitosan (CHI, polycation) and hyaluronic acid (HA, polyanion). The surface coating is built of a base-layer comprising 25 HA/CHI bilayers (BL) and a final HA layer to provide a negative charge for LPX adsorption. A cover layer composed of HA and CHI terminates the LPX-loaded PEM coating. (B) Lipid structures of the LPX components, OH4 and DOPE.

## 2 Materials and Methods

### 2.1 Materials

If not stated otherwise, the chemicals were acquired from Merck KGaA (Darmstadt, Germany). The phospholipid 1,2-di­(*9Z*-octadecenoyl)-*sn*-glycero-3-phosphoethanolamine (DOPE) was obtained from Avanti Polar Lipids (Alabaster, USA). The ionizable lipid *N*-{6-amino-1-[*N*-(9*Z*)-octadec-9-enyl-amino]-1-oxohexan-(2S)-2-yl}-*N′*-{2-[*N,N*-bis­(2-aminoethyl)­amino]­ethyl}-2-hexadecylpropandiamide (OH4) was synthesized in our research group.^19^ The plasmid DNA (pDNA, 3.5 kbp, 1 mg/mL stock, product abbreviation pCMV-GFP), encoding the green fluorescent protein, was purchased from PlasmidFactory (Bielefeld, Germany).

### 2.2 Polyelectrolyte Solutions

Sodium acetate buffer pH 5.5 was prepared by combining 0.2 M sodium acetate (Merck, Darmstadt, Germany), glacial acetic acid (AppliChem, Darmstadt, Germany) and water to a final concentration of 0.1 M acetate. The polyelectrolytes sodium hyaluronate (HA, *M*_w_ ≈ 1.3 MDa; Kraeber & Co GmbH, Ellerbek, Germany) and chitosan 85/500 (CHI, *M*_w_ ≈ 0.2–0.4 MDa, degree of deacylation ≈ 85%; Heppe Medical Chitosan GmbH, Halle (Saale), Germany) were separately dissolved in 0.1 M acetate buffer to a concentration of 2 mg/mL by stirring overnight and subsequent filtration using Minisart high flow syringe filters (4,4′-Dichlorodiphenyl sulfone-4,4′-dihydroxydiphenyl sulfone copolymer, pore size 0.45 μm; Sartorius, Göttingen, Germany).

### 2.3 Base-Layer PEM Preparation

Silicon wafers (10.0 × 15.0 mm, 0.6 mm thick, (100) face, Si-Mat, Kaufering, Germany) were used as substrates for the PEM base-layer preparation. The wafers were cleaned using a RCA-1 protocol^16^ and kept in Milli-Q water not exceeding 6 days. For loading efficiency determination, glass coverslips with a diameter of 13 mm and a thickness of 0.13–0.16 mm (Karl Hecht GmbH & Co KG, Sondheim vor der Rhön, Germany) were used as substrates. For automated PEM coating of the substrates, a DR 0 Layer-by-Layer deposition robot (Riegler & Kirstein GmbH, Potsdam, Germany) controlled by Dipp3dWin software was used. To fix the substrates for the dipping process, custom designed holders were utilized.^17^ Briefly, PEM preparation was performed by alternating incubation of the substrate in the polyelectrolyte solutions following the sequence: 5 min of HA solution, 2.5 min washing step in acetate buffer pH 5.5, 5 min of CHI solution, 2.5 min washing step in acetate buffer pH 5.5. This sequence was repeated until the base-layer PEM [HA, CHI]_25_HA was completed. The preformed base-layer PEMs were stored separately in a 12-well plate in 0.1 M acetate buffer pH 5.5 at 4 °C until used for measurements or further processing. The process is schematically illustrated in Figure 2A.

**Figure 2:**
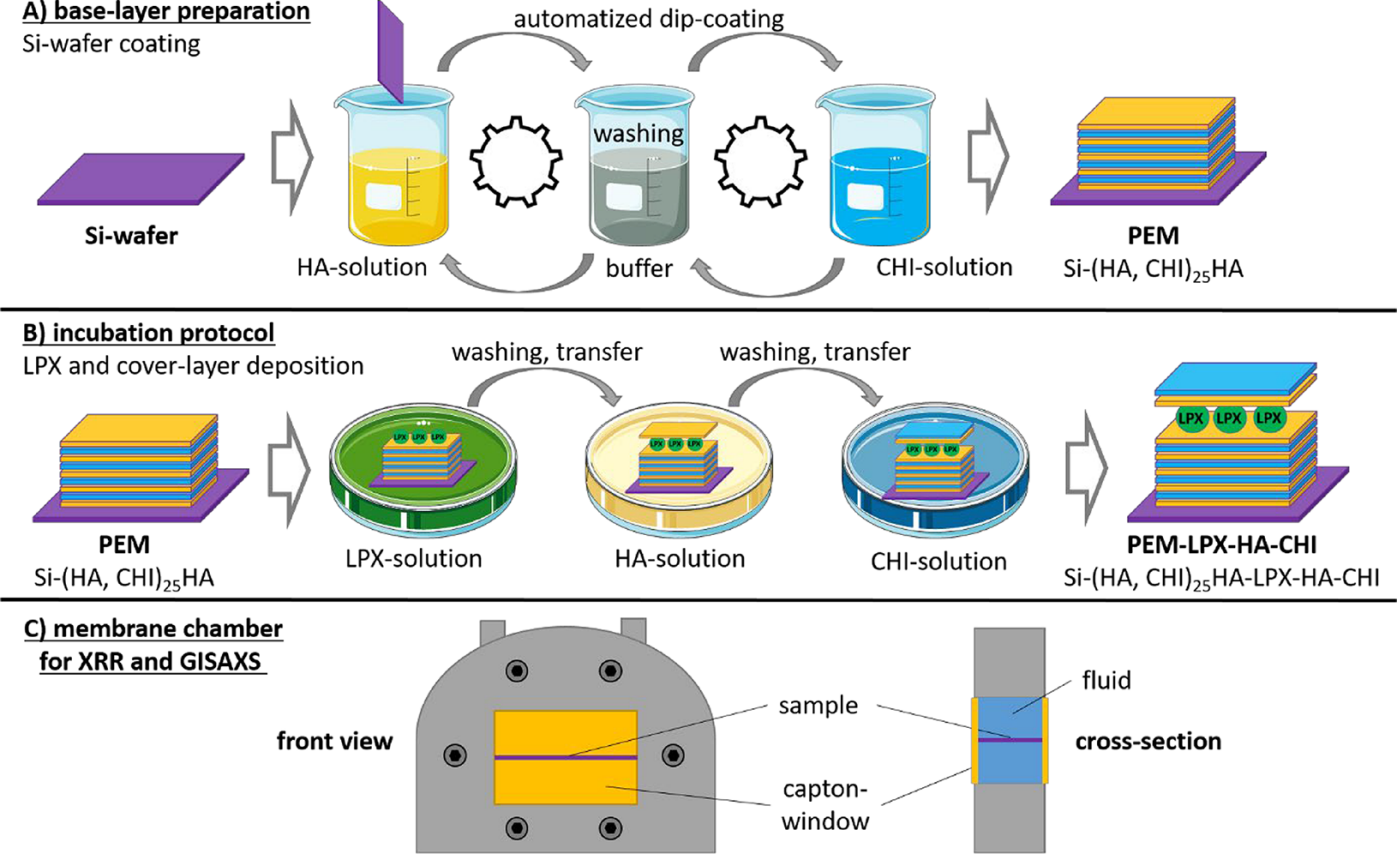
Illustration of the coating process applied to the silicon wafers (panel A). The base-layer PEM (sequence: Si-(HA, CHI)_25_HA was prepared by using an automated dip coating protocol. The LPX deposition and coverlayer adsorption was performed by an incubation protocol in 12-well plates (panel B). Panel C displays the polyether ether ketone (PEEK) chamber body for placing the coated silicon wafer in aqueous environment, with the Kapton foil windows, used for synchrotron X-ray experiments. The two Kapton windows were clamped by two Aluminum window frames (not shown) from both sides of the chamber body. The frames consisted of water channels for temperature control. A Pt100 temperature sensor (not shown) was embedded into the PEEK body in contact with the aqueous solution.

### 2.4 Liposome Preparation

OH4 and DOPE were separately dissolved in chloroform/methanol (8/2 v/v) to a concentration of 2 mg/mL and mixed in a final molar ratio of 1:1. The organic solvent was removed using a rotary evaporator at 500 mbar for 30 min, followed by 10 mbar for 1.5 h. The dried lipid film was resolvated in 0.1 M acetate buffer pH 5.5 by shaking at 600 rpm for 30 min at 55 °C using an Eppendorf ThermoMixer C (Eppendorf SE, Hamburg, Germany). To form liposomes, sonication at 55 °C and 37 kHz for 15 min using an Elmasonic P sonication bath (Elma Schmidbauer GmbH, Singen, Germany) was performed. The final total lipid concentration in the liposome dispersion was 1 mg/mL. Liposomes were stored at 4 °C until use, and sonication at room temperature was conducted for 5 min right before LPX preparation.

### 2.5 Lipoplex Preparation

For LPX preparation at an N/P ratio of 4 (ratio of primary amines of OH4 - N - to phosphate groups of the nucleic acid, P), 5.85 μL of the pDNA solution (1 mg/mL) and 39.89 μL of the liposome dispersion (1 mg/mL) were diluted to 70 μL each in 0.1 M sodium acetate buffer at pH 5.5. The obtained pDNA dilution was added to the liposome dispersion in one step and mixed by gentle pipetting, followed by shaking for 15 min at 300 rpm with a ThermoMixer C apparatus (Eppendorf SE, Hamburg, Germany). Multiple batches were then combined and blended to obtain a sufficient quantity of LPX to load the PEMs. The procedure of combined batches was necessary, and the LPX production was validated for the small volumes mentioned above, and test volume upscaling resulted in larger LPX.

### 2.6 Lipoplex Deposition on Base-layer PEMs - Incubation Protocol

LPXs were diluted to “LPX-loading dispersions” of the following DNA concentrations: 4.33 ng/μL (1 × c­(LPX)), 8.66 ng/μL (2 × c­(LPX)), 17.32 ng/μL (4 × c­(LPX)) and 34.64 ng/μL (8 × c­(LPX)). For LPX loading of base-layer-PEMs an incubation protocol was used (see schematic illustration in Figure 2B). Base-layer PEMs were incubated with 1050 μL of the appropriate LPX-loading dispersion in a 12-well plate for 2 h on a 3D rocker shaker (VWR International GmbH, Darmstadt, Germany) at speed/tilt 8. Afterward, PEMs were washed twice with buffer for 5 min. If a deposition of HA and CHI coverlayers was applied, the PEMs were subsequently incubated for 10 min with 1050 μL of HA solution, washed twice with buffer, and afterward incubated for 10 min with 1050 μL of CHI solution and finally washed twice with buffer. All steps were conducted on a 3D rocker shaker.

### 2.7 Synchrotron X-ray Experiments

GISAXS and XRR measurements were used to examine the change of the LPX mesostructure and the structure of the PEM coating upon the embedding process. The silicon wafers, either PEM-coated or noncoated (depending on the experiment), were mounted into the SDU-Odense membrane chamber (AG. Klösgen, University of Southern Denmark, Denmark) (Figure 2C). The wafers were inserted centrally and horizontally and tightly sealed in the chamber. Through a side port, 1.5 mL of the acetate buffer was filled into the compartment for the measurement buffer (≈500 μL below and ≈1000 μL above the wafer). The chamber was mounted in the experimental setup and then thermostatically adjusted to 22.0 ± 0.2 °C for about 10 min prior to the X-ray measurement. The real-time temperature of the aqueous phase was monitored over preparation and measurement by an integrated Pt100 sensor in the chamber.

For most of the measurements, the PEM coating (with or without LPX embedding) was performed ex-situ before mounting the silicon wafer in the chamber. For the in situ evaluation of LPX deposition on the silicon wafers with base-layer PEM coating or empty silicon wafers, the LPX deposition was performed in the liquid chamber. 500 μL of the buffer were removed from the chamber and replaced by 500 μL of a LPX dispersion at a DNA concentration of 33.3 ng/μL, resulting in a nominal concentration of ≈17 ng/μL. During this loading process, the wafer was always immersed in the buffer.

The measurements were conducted on a Kohzu 6-circle diffractometer at the beamline P08 of the PETRA III synchrotron (DESY, Hamburg Germany).^20^ An incident beam at 18 keV with focus mode was used, providing a beam size of 0.07 mm × 0.3 mm (vertical × horizontal). A set of guard slits and a presample pinhole with a diameter of 0.8 mm were used for reducing the beam path scattering background before the sample. The XRR signal was measured with a Pilatus 100k (Dectris, Switzerland) at 1000 mm from the center of the sample surface, positioned at two times the sample incident angle with respect to the incident beam (θ–2θ geometry). The reflected intensity was integrated in a detector area of 1.0 mm × 1.5 mm (vertical × horizontal) and normalized to the incident beam intensity. The GISAXS signal was measured with an Eiger2X 1 M detector (Dectris, Switzerland) placed 1218 mm from the sample surface center. The direct and the reflected beams were blocked by a 1.2 mm wide Tungsten finger beamstop placed at ≈300 mm from the sample. The angle of incidence with respect to the sample plane was set as a series of 0.07, 0.2, and 0.5°. Note that the lowest angle corresponds to a total-reflection condition of the silicon surface (critical angle: ∼0.095°). The buffer GISAXS background signal of the chamber setup was measured by lowering the sample by 1 mm to be subtracted from the sample surface scattering signal. Azimuthal integration of the GISAXS data was performed using pyFAI library^21^ to obtain one-dimensional (1D) GISAXS diffractogram for further analysis.

### 2.8 Small Angle X-ray Scattering

For SAXS measurements, the samples were prepared by modifications from the LPX preparation protocol to achieve higher concentration, details are described in the Supporting Information (chapter 1, SAXS sample preparation). All samples were transferred in borosilicate glass capillaries which were sealed afterward (1.5 mm outside diameter, 0.01 mm wall thickness; WJM-Glas/Müller GmbH, Berlin, Germany). The SAXS measurements were carried out at the Biomaterials Department of Max Planck Institute of Colloids and Interfaces (MPIKG, Potsdam, Germany) with a Bruker Nanostar 2 (Bruker, Billerica, MA, USA), equipped with a 2D Vantec-2000 detector and a microfocus X-ray source (*I* μS) with 1.542 Å wavelength (Cu Kα) and a focal spot size of 115 μm. The following measurement parameters were set: 50 kV voltage, 600 μA current, 1071 mm sample–detector distance, and the *q* resolution was characterized to be 0.007/Å (Gaussian half-width-at-half-maximum). As calibration material, silver behenate was used. The capillaries were placed in a sample holder and the total scanning time was 85 h. For data processing SAXS: Small Angle X-ray Scattering System V4.1.61 software (Bruker, Billerica, MA, USA) was used. As background measurements, capillaries filled with buffer, empty capillaries, and the empty sample holders were measured (Supporting Information, Figure S4).

### 2.9 Quantification of the DNA Loading

The pDNA loading of PEMs was quantified indirectly by determination of pDNA concentration in the supernatant (only indirect quantification was applicable on this system, see explanation in Husteden et al.^16^). Briefly, the supernatant and washing solutions were collected after PEM incubation with the LPX-loading dispersion. The samples of the washing solutions were combined for analysis. For total pDNA quantification, a disintegration/decomplexation of LPX was necessary. For this purpose, 150 μL of the collected solutions were transferred into microvessels and mixed with a 60 mg/mL heparin solution (sodium heparin from porcine intestinal mucosa, ≥180 USP units/mg) (Sigma-Aldrich, Taufkirchen, Germany) in Dulbecco’s PBS (AppliChem, Darmstadt, Germany) to a final concentration of 2 μg/μL heparin in the samples. The samples were shaken for 5 min at 1500 rpm in a ThermoMixer C apparatus (Eppendorf SE, Hamburg, Germany). Subsequently, an equal volume of isopropanol (solvent temperature 0 °C) was added to the samples and incubated for 20 min at −20 °C to precipitate the pDNA. After centrifugation at 21,300*g* for 10 min at 4 °C, the solvent was carefully removed from the samples. 500 μL of ethanol (solvent temperature 0 °C) was used to wash the resulting pellet. After removal of ethanol, the samples were resuspended in 20 μL of water (pH 8). To quantify the pDNA amount, a digestion was implemented to linearize the plasmid. 10 μL of the DNA solution were separately mixed with 7.6 μL water, 2 μL of rCutSmart Buffer and 0.4 μL XhoI (both from New England Biolabs, Ipswich, MA, USA) restriction endonucleases. The mixture was incubated for 1 h at 37 °C. As a standard for quantification, pDNA was treated the same way. Furthermore, TriTrack DNA Loading Dye (6×) (Thermo Fisher Scientific, Darmstadt, Germany) was added, and the samples were applied to agarose gels. The gels were prepared in a concentration of 1% agarose in TAE buffer with thiazol-orange. The GeneRuler 1 kb Plus DNA ladder (Thermo Fisher Scientific, Darmstadt, Germany) and pDNA in different concentrations for the calibration curve for assessment were also loaded onto the gel. The electrophoresis was performed for 1 h at 120 V and the gels were evaluated using a GelDoc Go Imaging System (Bio-Rad Laboratories, Hercules, USA). The gel is shown in the Supporting Information (Figure S33).

### 2.10 Determination of the Thickness Values of the Bilayer Lamellae

The lamellar repeat distance *d* was calculated from the peak maximum of a Bragg reflection as *d* = (2*n*·π)/*q*_*n*_, where *q*_*n*_ is the magnitude of the scattering vector and *n* is the order of the Bragg reflection. The calculated *d* values are given as mean for 3 different positions in the capillary (SAXS) or 4 different positions on the silicon wafer (GISAXS). For SAXS and GISAXS analysis, the peaks were fit by Lorentzian functions to obtain the center using OriginPro 2019 software (representative examples are presented in SI Figures S5–S7, S19, S21, S23, S25–S32, and S34–S36). The intensity *I*(*q*_*n*_) of the *n*-th order SAXS peaks were also used for a qualitative Fourier reconstruction to yield the head-to-head thickness *d*_HH_ of the bilayers.^22^ In the first step, the form factor values *F*(*q*_*n*_) at the positions *q*_*n*_ of the *n*-th order Bragg peaks were calculated as $F(q_{n})=\sqrt{q_{n}^{2}I(q_{n})}$ where *q*_*n*_^2^ is the Lorentzian factor correction for unoriented membrane stacks. We calculated a series of form factor values *F*(*q*_3_) by assuming that the intensity *I*(*q*_3_) is between 5 and 100% of *I*(*q*_2_), a typical range for lecithin lamellae above 80% hydration.^23^ Note that three Bragg peak orders are required and sufficient to determine *d*_HH_.^22^ Also note that the structure factor correction of lamellae-disorder induced diffuse scattering is not necessary for this purpose, especially at the applied broad resolution.^22^ The electron density contrast Δρ_e_(*z*), albeit at low resolution, was calculated as

$$\Delta \rho_{e}(z)=\sum_{n=1}^{3}\{\pm \}F(q_{n})\cos\left(\frac{2n\pi z}{d}\right)$$

where {±} is the phase factor (−, −, +) at the first, second and the third order peak positions for lecithin^23^ (see also Supporting Information Figures S8–S10). *d*_HH_ was obtained from the distance between the two maxima in Δρ_e_(*z*), and its uncertainty was the half-range of its variation from the intensity assumption of the third order peak. The interbilayer distance *d*_int_ = *d* – *d*_HH_ was calculated for both SAXS and GISAXS data, and for latter the *d*_HH_ was assumed to be the same as that of the corresponding SAXS sample.

## 3 Results and Discussion

### 3.1 General Characteristics of LPX

The used OH4/DOPE N/P 4 LPX were prepared via complex formation between cationic liposomes composed of OH4/DOPE 1/1 (*n*/*n*) (unilamellar character due to the positive charge, demonstrated via cryo transmission electron microscopy and SAXS^18^) and pDNA at a lipid amino group/DNA phosphate (N/P) ratio of 4. The average size of OH4/DOPE N/P 4 LPX was determined to be ≈200 nm by dynamic light scattering. The zeta potential was ≈ +40 mV. This positive charge is a requirement for the deposition of the LPX on PEMs with HA as the terminal polyelectrolyte layer. Further details on the LPX characteristics can be found elsewhere.^17,24^

### 3.2 SAXS Measurements on LPX in Dispersion

SAXS measurements were performed to identify the mesophase substructure of OH4/DOPE N/P 4 LPX nanoparticles in acetate buffer (100 mM, pH 5.5). The same buffer system has been used for embedding of LPX in polysaccharide PEMs.^17^ Earlier studies of OH4/DOPE N/P 4 LPX in MES buffer (100 mM, pH 6.5) reported a lamellar L_α_^c^ LPX structure (Figure 3B), a supermolecular multilamellar mesophase structure with alternating lipid bilayer and DNA monolayers in the interlamellar aqueous space^4^ with lamellar repeat distance *d* = 69 Å.^18^ For OH4/DOPE N/P 4 LPX in acetate buffer (100 mM, pH 5.5) the Bragg peaks also indicated a lamellar L_α_^c^ LPX structure showing the first and second order peak, at *q*_1_ and *q*_2_ = 2*q*_1_, respectively (see Figure 3A and Table 1). The determined *d* value (see schematic illustration in Figure 3B) ranged between 64.2 and 66.6 Å depending on the preparation procedure of samples 1–3 (three different procedures were chosen to achieve LPX preparations with high concentrations). A significantly lower *d* value was observed compared to the LPX in MES buffer at pH 6.5, and we attribute this difference to the difference in the charge states under the two pH conditions. In an earlier study, we determined the apparent p*K*_a_ values (p*K*_a^app^_) of homologue OH4 derivatives to be p*K*_a^app^_ ≈ 6 by quantification of counterion adsorption to the headgroup-water interfacial region and thus the protonation degree of the ionizable amines.^25^ Although recent research demonstrated, that the protonation degree can differ significantly from the value expected from the Henderson–Hasselbalch equation,^26^ we want to use the above-mentioned p*K*_a^app^_ of 6 for theoretical consideration. Calculating the ratio of R-NH_3_^+^/R-NH_2_ using the Henderson–Hasselbalch equation [pH = p*K*_a_ – lg­(*c*{R-NH_3_^+^}/*c*{R-NH_2_})] and a p*K*_a_ of 6 results in a R-NH_3_^+^/R-NH_2_ ratio of 0.32 for MES pH 6.5 and 3.16 for acetate pH 5.5. Consequently, the charge density of the lipid formulation is higher in the acetate buffer. An increase of positive charge density of the lipid formulation caused by a higher protonation degree can cause a decrease in the *d* value of L_α_^c^ LPX due to a stronger electrostatic interaction with the negatively charged DNA between the lamellae.^27–29^ The interbilayer distance *d*_int_, i.e., the space for the embedded DNA, was found to be 20.3–22 Å, as calculated from the head-to-head thickness *d*_HH_ of 43.9–44.2 Å (see Table 1). This value is consistent with the 20 Å diameter of DNA assuming a rod structure.^30^

**Figure 3:**
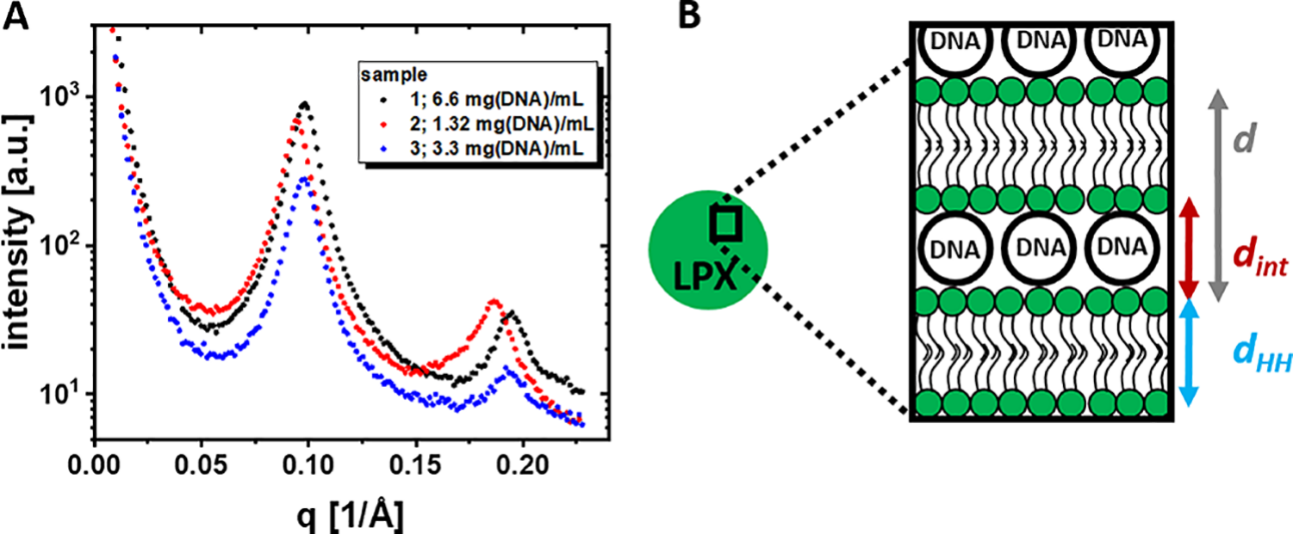
(A) SAXS pattern of OH4/DOPE N/P4 LPX in acetate buffer pH 5.5. The three different samples were prepared by different protocols and resulted in different final LPX concentrations. (B) Illustration of the L_α_^c^ LPX structure.

**Table 1:** Summary of SAXS Results of OH4/DOPE NP4 LPX in Acetate Buffer at pH 5.5 for the Three Different Samples

	sample 1	sample 2	sample 3
*c*_LPX_ ^a^ [mg/mL]	6.6	1.32	3.3
*q*_1_ ^b^ [1/Å]	0.098 ± 3 × 10^–5^	0.094 ± 3 × 10^–5^	0.097 ± 3 × 10^–5^
*q*_2_ [1/Å]	0.194 ± 8 × 10^–5^	0.186 ± 3 × 10^–4^	0.193 ± 3 × 10^–4^
*q*_2_ ^c^ calculated	0.196	0.189	0.195
*d* ^d^,^b^ [Å]	64.15 ± 0.02	66.6 ± 0.02	64.5 ± 0.02
*d*_HH_ [Å]	43.9 ± 0.7	44.7 ± 0.7	44.2 ± 0.9
*d*_int_ [Å]	20.3 ± 0.7	22.0 ± 0.7	20.3 ± 0.9

a Calculated for DNA content.

b Mean ± standard deviation determined by Lorenz-fits of the 3 different curves, given in the Supporting Information Figures S1–S3 and Tabe S1).

c Calculated as 2·*q*_1_

d *d* = 2π/*q*_1_

### 3.3 Characterization of LPX Embedded in Polysaccharide PEMs

For LPX embedded in PEMs composed of HA and CHI, the chemical environment of the lipid nanoparticles changes drastically compared to the dispersion of the nanoparticles in aqueous medium. Hence, LPX supermolecular assembly is based on electrostatic interaction and entropic effects.^31,32^ It is thus plausible to expect structural changes of the LPXs caused by the embedding, and the characterization of such changes is the central aspect of the present work.

GISAXS was used to screen the surface coating for structural features. The azimuthally integrated GISAXS data of the functionalized base PEM (sequence on the silicon wafer: Si-(HA, CHI)_25_HA, Figure 4D/E, curve labeled PEM) shows a curve with monotonic intensity decay and the absence of Bragg reflection peaks. The deposition of LPX using the standard LPX loading-dispersion we also used for transfection experiments in earlier work^17^ (LPX-loading dispersion concentration 4.33 ng/μL) and addition of the cover layer (sequence on the silicon wafer: Si-(HA, CHI)_25_HA-LPX-HA-CHI) changes the diffractogramm (Figure 4D/E, curve labeled 1*c*(LPX)). The sample shows a distinct Bragg peak that is the first order reflection (indicated as *q*_1_) of the L_α_^c^ LPX structure. The embedding into PEM decreases the spacing in LPX by 7 to 9 Å to *d* ≈ 57 Å, suggested by the shift of peak position *q*_*n*_ to higher values compared to the SAXS results for LPX in dispersion (Table 1 vs Table 2). In the following, this L_α_^c^ LPX structure with lower *d* values is referred to as L2-LPX. Evaluation of the Bragg signal of the baseline-corrected curve by fitting two Lorentzian functions indicate the presence of an additional broad peak associated with *d* values more comparable to LPX in dispersion (see Supporting Information Figure S34). This structure will be termed L1-LPX in the following. The ratio of the L1 to L2 peak intensities was 2.5 ± 1.2. Note, that LPX embedded in PEMs prepared with the 1 × *c*(LPX) and stored for 4–5 days in buffer still exhibit the distinct signal of the L2-LPX phase (see Supporting Information chapter 4 and Figures S14–S17). This leads to the questions of whether it is possible to embed higher amounts of LPX into the PEM structure and whether or not the ratio of the two different lamellar LPX phases is affected. We increased the concentration of the LPX-loading dispersion for the LPX-deposition by factors 2 and 4 (samples labeled as 2 × *c*(LPX) and 4 × *c*(LPX)). Evaluating the effectivity of LPX embedding in this set of experiments, the LPX loading given as amount DNA normalized to the surface was significantly increased with the increase of the concentration of the LPX-loading dispersion in the screened range (Figure 4B). Hence, the possibility of incorporating higher amounts of LPX into the PEM was demonstrated. The diffractogram also changes with increasing LPX mass deposited (Figure 4D/E). With an increasing amount of LPX loading, the peak signal increases in intensity. The area of the signal is proportional to the volume fraction of the LPX in the measurement area of the sample in the GISAXS setup. Consequently, a higher peak area corresponds a higher amount of embedded LPX. The second observation was a clear appearance of the two different first order peaks of the two lamellar LPX structures for the 2 × *c*(LPX) and 4 × *c*(LPX) sample (see reflex labeled L1-LPX and L2-LPX in Figure 4D/E and Table 2). The peak at *q* ≈ 0.10 Å^–1^ was assigned to L1-LPX and the peak at *q* ≈ 0.11 Å^–1^ was assigned to the L2-LPX phase, as described above for the sample 1 × *c*(LPX). The L1/L2 intensity ratios of the two Bragg peaks were 1.5 ± 0.9 for 2 × *c*(LPX) and 25 ± 17 for 4*c*(LPX) (details of peak area determination in the Supporting Information chapter 7). Apparently, at 4 × *c*(LPX) the proportion of the L2-LPX phase could not increase further, and the L1-LPX phase becomes dominant, as reflected by the high L1/L2 ratio. Note that it is not possible to quantify the amount of deposited LPX from the peak intensity due to the high PEM background and inhomogeneous loading concentration on the centimeter-large surface (Tables S4 and S5). Further it was planned to increase the concentration of the LPX-loading dispersion in the LPX-deposition by a factor of 8 (sample 8 × *c*(LPX)), but a visible aggregation of LPX on the PEM was observed. This aggregation also resulted in a very inhomogeneous distribution of LPX on the PEM, as demonstrated by pronounced variations of the signal intensities in the GISAXS position scan (Supporting Information, Figure S24). Due to the high inhomogeneity, these high loading concentrations should be avoided for the application of the LPX-loaded PEMs.

**Figure 4:**
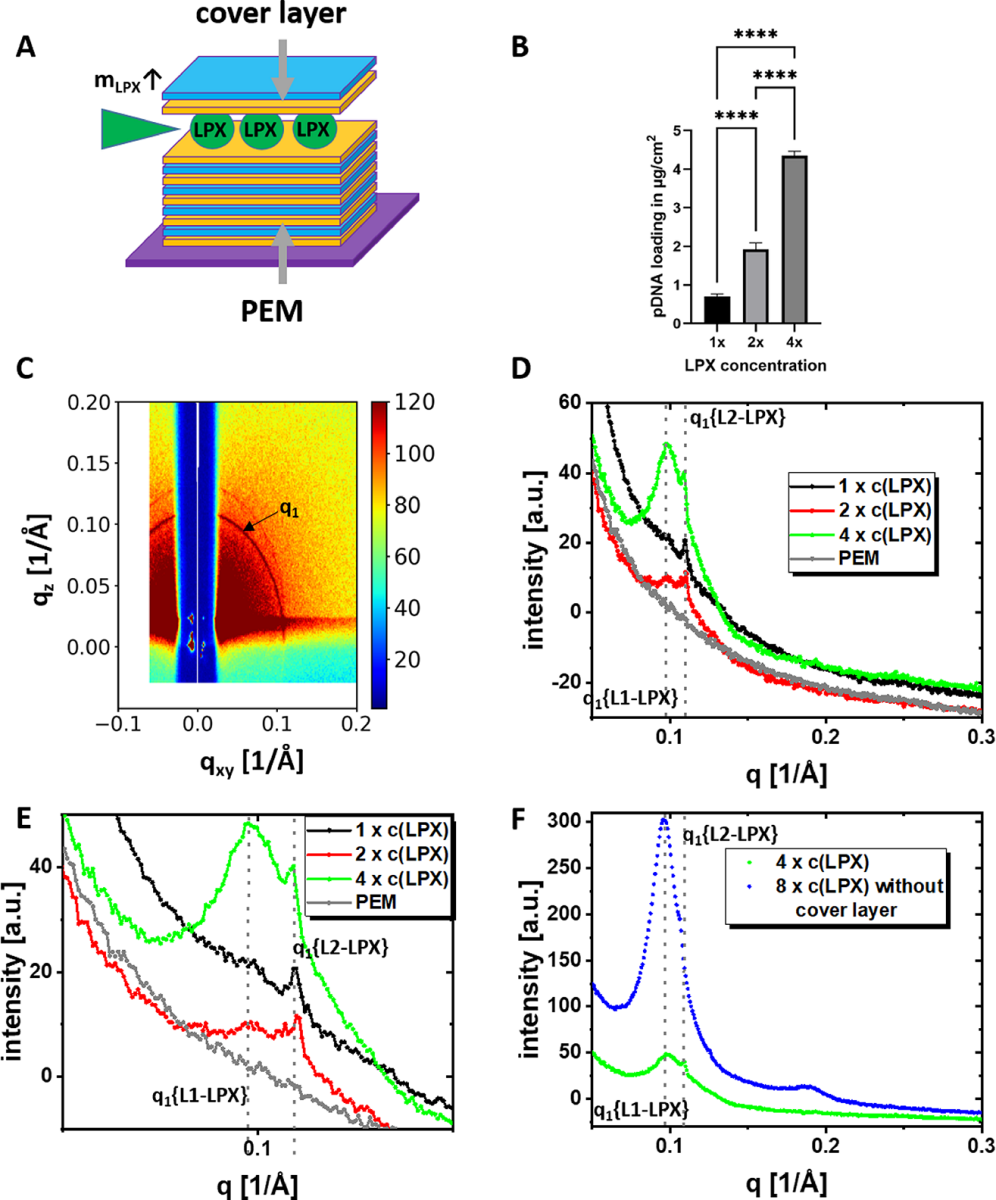
(A) Schematic illustration of the investigated LPX-loaded PEMs. The mass of embedded LPX was increased by performing the LPX deposition with an increased LPX concentration (c­(LPX)) in the LPX loading dispersions. (B) Determination of loading efficiency of PEM-films with LPX cargo by indirect DNA quantification using gel electrophoresis given in absolute DNA amount in g/cm^2^ for PEMs loaded with the LPX loading dispersion single, double and quadruple concentration (labeled with 1×, 2×, and 4×). Given values are mean ± standard deviation of *n* = 3. One way anova with Tukey post hoc test was performed to check for statistical significance (**** = *p* < 0.0001). C–F) GISAXS experiments of Si-(HA, CHI)_25_HA-LPX-HA-CHI samples using different LPX concentrations in the LPX deposition step. (C) example 2D GISAXS pattern before azimuthal integration. The first order peak of the L_α_^c^ LPX structure is labeled as *q*_1_, and has the form of a Scherrer ring. (D) Azimuthally integrated 1D GISAXS diffractogram of PEMs with different LPX loading concentrations. The *q*_1_ reflexes of two different lamellar phases were observed, labeled with *q*_1_ {L1-LPX} and *q*_1_ {L2-LPX}. The concentration of the LPX dispersion for the LPX loading were varied from the standard concentration 1 × *c*(LPX) to higher concentrations, namely 2 ×, and 4 × (representing the factor of concentration increase). (E) Detailed *q* range from (D). (F) GISAXS experiments of Si-(HA, CHI)_25_HA-LPX-HA-CHI with 4 × *c*(LPX) and Si-(HA, CHI)_25_HA-LPX with 8 × *c*(LPX) loading. The GISAXS diffractograms of the samples presented in (D), (E), and (F) are representatives of scans at 4 different positions on the sample given in the Supporting Information Figures S18, S20, S22, S24.

**Table 2:** LPX Bragg Peak Position *q*_1_ Obtained From GISAXS Measurement of OH4/DOPE N/P 4 LPX on or Embedded in PEMs as Mean of 4 Different Curves at Different Positions and the Calculated *d* Value as Well As The *d*_int_ Value^a^

sample	parameter	L1-LPX	L2-LPX	cover layer	*c*_LPX_ ^b^ [μg/mL]
1 × *c*(LPX)	*q*_1_ ^c^ [1/Å]	0.1025 ± 0.0035	0.1096 ± 0.0004	yes	4.33
	*d* [Å]	61.3 ± 2.1	57.3 Å ± 0.2		
	*d*_int_^min^ [Å]	16.6 ± 0.7	12.6 ± 0.7		
	*d*_int_^max^ [Å]	17.4 ± 0.7	13.4 ± 0.7		
2 × *c*(LPX)	*q*_1_ ^c^ [1/Å]	0.1016 ± 0.0006	0.11 ± 0.0001	yes	8.66
	*d* [Å]	61.8 ± 0.4	57.1 ± 0.1		
	*d*_int_^min^ [Å]	17.1 ± 0.7	12.4 ± 0.7		
	*d*_int_^max^ [Å]	17.9 ± 0.7	13.2 ± 0.7		
4 × *c*(LPX)	*q*_1_ ^c^ [1/Å]	0.0992 ± 0.0003	0.1092 ± 0.0001	yes	17.32
	*d* [Å]	63.4 ± 0.2	57.6 ± 0.1		
	*d*_int_^min^ [Å]	18.7 ± 0.7	12.9 ± 0.7		
	*d*_int_^max^ [Å]	19.5 ± 0.7	13.7 ± 0.7		
8 × *c*(LPX)	*q*_1_ ^c^ [1/Å]	0.0961 ± 0.0003	0.1063 ± 0.0001	no	34.64
	*d* [Å]	65.4 ± 0.2	59.1 ± 0.1		
	*d*_int_^min^ [Å]	20.7 ± 0.7	14.4 ± 0.7		
	*d*_int_^max^ [Å]	21.5 ± 0.7	15.2 ± 0.7		

a *d*_int_ values are calculated from the highest *d*_HH_ value (*d*_int_^min^) and the lowest *d*_HH_ value (*d*_int_^max^) of Table 1.

b Concentration of 1050 μL of the LPX loading dispersion calculated for DNA content.

c Mean ± standard deviation of Lorenz-fits of the 4 different scanning positions, given in the Supporting Information Table S3).

After two different lamellar LPX structures were observed, the process of LPX deposition was screened in situ (see Figure 5) to get more insights in the process of deposition. The concentration of LPX in the chamber was comparable to the 4 × *c*(LPX) experiment mentioned above. Within the screened time frame of 540 min, the intensity of the first order peak of the two lamellar LPX phases increased with increasing time (Table 3), which can be attributed to an increasing amount of adsorbed L1- and L2-LPX. Hence, the adsorption of LPX on the PEM was efficient, and it is possible to increase the deposited amount of LPX by the increase of incubation time. The driving force of the adsorption can be attributed to the electrostatic interaction of the LPX (positive zeta potential^17,24^ ) with the negatively charged HA of the basal PEM. Further, the in situ experiment revealed the coexistence of the two structural LPX types (L1-LPX and L2-LPX, Figure 5 and Table 3). Apparently, the adsorption of the coverlayer is not necessary for the formation of L2-LPX (detected for the 1 × *c*(LPX), 2 × *c*(LPX) and 4 × *c*(LPX) samples which had a coverlayer; but also, for the in situ deposition and the 8 × *c*(LPX) experiment without coverlayer; see Figures 4 and 5). Instead, the one-sided contact of the LPX with polyelectrolyte layers seems sufficient to trigger L2-LPX formation. Further, the in situ deposition experiment shows that the L1/L2 ratio increases significantly at long deposition times (Table 3), with values ranging between 2.4 and 3.8 within the first 180 min, but reaching 5.6 after 540 min. Nevertheless, the question arises if the effect of the appearing L2-LPX structure is generally caused by the LPX adsorption on surfaces. Hence, we performed an in situ experiment of LPX adsorption to the bare silicon wafer for 2 h (Figure 5D/E). Also here a certain affinity of the LPX to the surface was observed by detecting Bragg reflections. The signal intensity was less compared to the in situ adsorption to PEM coated silicon for the same adsorption time, demonstrating the relevance of the electrostatic interaction between the terminal HA PEM layer and the LPX as the driving force of the LPX adsorption process on the surface. Further, no signal of L2-LPX was observed for the LPX adsorption on silicon, indicating that the contact to the PEM is needed for the transition.

**Figure 5:**
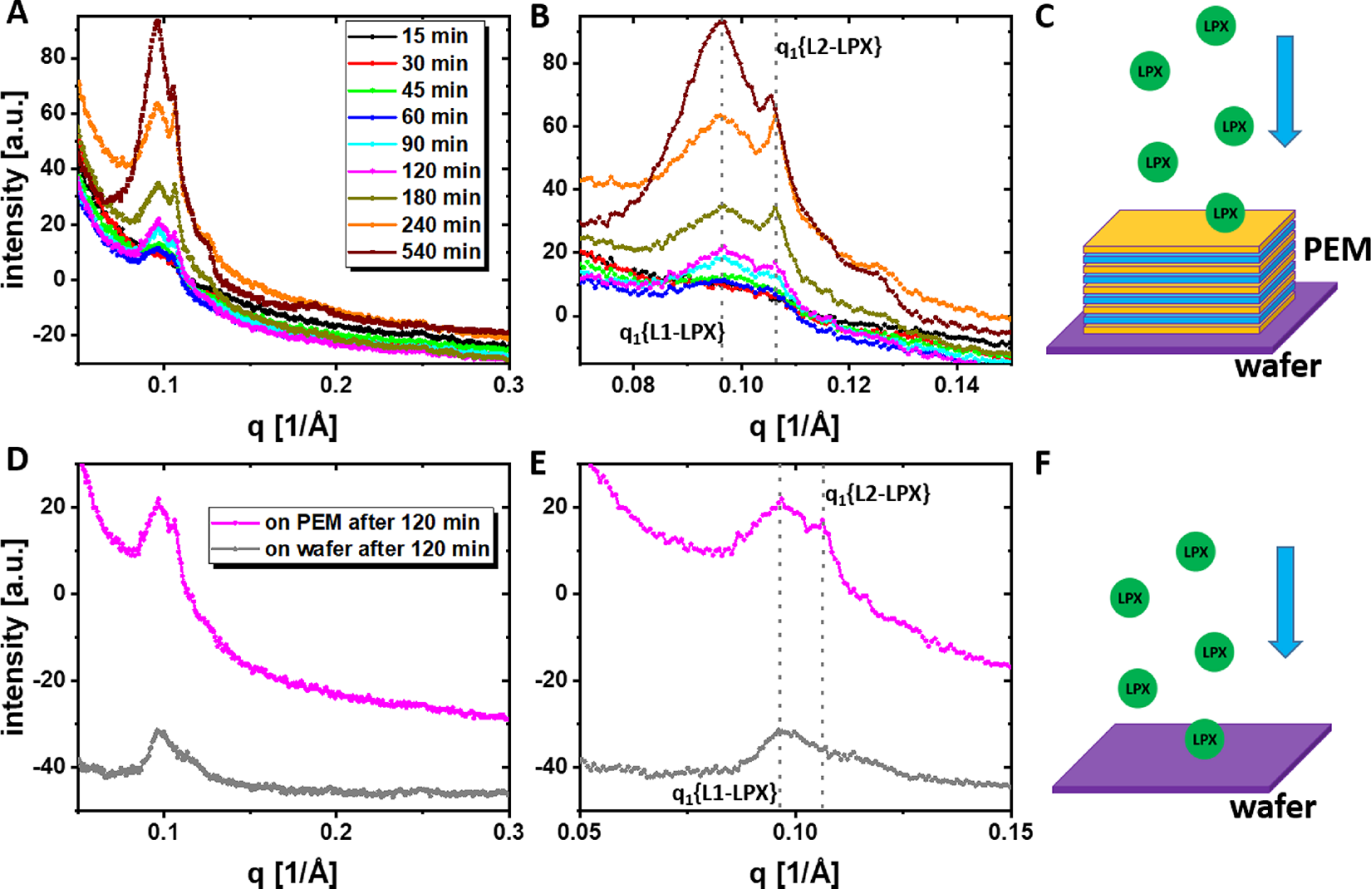
GISAXS in situ LPX deposition experiments of PEMs Si-(HA, CHI)_25_HA or unmodified silicon wafers with LPX in the supernatant for sedimentation. In the liquid above the modified or unmodified silicon wafer, the LPX concentration was 17 ng_(DNA)_/μL. (A) 1D GISAXS diffractogram of Si-(HA, CHI)_25_HA PEMs adsorbing LPX from solution after different time points in the LPX containing medium. The two different lamellar phases were labeled with *q*_1_ {L1-LPX} and *q*_1_ {L2-LPX}. (B) Detailed *q* range from A. (C) Schematic illustration of the experiment based on free sedimentation and adsorption of LPX on the basal PEM. (D) 1D GISAXS diffractogram of silicon wafer deposited LPX compared to Si-(HA, CHI)_25_HA PEMs deposited after an adsorption period of 120 min in the in situ deposition experiment. (E) Detailed *q* range from D. (F) Schematic illustration of the experiment based on free sedimentation and adsorption of LPX on the bare silicon wafer.

**Table 3:** Calculated *d* Values and Peak Areas Determined via Lorentz Fit From GISAXS Measurement of OH4/DOPE N/P 4 LPX In-situ Deposition on PEM with a Starting Concentration of 17 ng/μL LPX (Calculated for DNA) in the Supernatant^a^

	L1-LPX		L2-LPX		
time	*d* [Å]	area^b^ [a.u.]	*d* [Å]	area^b^ [a.u.]	L1/L2 ratio
45 min	63.6	0.22 ± 0.03	59	0.06 ± 0.02	3.8
60 min	65.3	0.22 ± 0.02	59.4	0.08 ± 0.01	2.6
90 min	64.3	0.29 ± 0.01	59.0	0.08 ± 0.01	3.5
120 min	64.7	0.37 ± 0.02	59	0.15 ± 0.02	2.4
180 min	64.6	0.50 ± 0.02	59	0.15 ± 0.01	3.3
240 min	64.9	0.87 ± 0.02	58.9	0.21 ± 0.01	4.2
540 min	65.7	1.96 ± 0.05	59.2	0.35 ± 0.01	5.6

a Every time value was scanned once at a different position. L1/L2 ratio is calculated from the area of the *q*_1_ peak of the individual lamellar phase. Peak fitting to determine the *d* values are shown in Figures S26–S32 and for area determination in Figures S37–S42.

b Mean ± error determined by Lorentz function fit.

We further performed XRR measurements to check whether or not the PEM coatings have a layered internal structure themselves, as described in literature,^33^ or if the nanoparticles cause structural features which can be detected by XRR. The reflectivity measurements indicate no relevant electron density modulation within the PEMs (for more detailed discussion see Supporting Information, chapter 8 and Figure S43).

### 3.4 General Discussion

The presented experiments demonstrate that LPX composed of the lipid mixture OH4/DOPE loaded with DNA to a N/P ratio of 4 can successfully adsorb to HA/CHI PEMs (in situ experiment, Figure 5A/B) and also be embedded in the PEM structure (Figure 4D/E). The PEM components HA and CHI are both weak polyelectrolytes which are loosely associated in a swollen PEM with high water content^34^ and, consequently, produce a surface coating with gel-like structure. The PEM-adsorbed LPXs seem to have no preferred orientation in the coating, indicated by the GISAXS pattern showing the Bragg reflection homogeneously distributed along the Scherrer ring (ring labeled *q*_1_ in Figure 4C). When in contact with the HA/CHI PEMs, the repeat distance *d* of a fraction of the lamellar LPX structure decreases by ≈7–10 Å compared to that of the LPX in dispersion. This observation needs to be discussed in more detail. The *d* value is the sum of the lipid bilayer thickness (*d*_HH_) and the interlamellar water layer thickness (*d*_int_) which also contains the DNA; (*d* = *d*_HH_ + *d*_int_). Hence, a smaller *d* value can result from decreased *d*_HH_ and/or *d*_int_. An effect of the LPX adsorption onto PEM on *d*_HH_ can be excluded as dominating factor for the increase since OH4 and DOPE as well as the mixtures are already in the liquid-crystalline phase state (high amount of *gauche*-conformers).^19,35^ Consequently, a reduced *d*_int_ value must be responsible for the observed phenomenon. Hypothetically, the loss of the nucleic acid cargo DNA from the LPX could explain a decrease in *d*_int_. This hypothesis can be withdrawn by the fact that experiments with fluorescently labeled LPX (lipid and DNA colabeling) embedded in HA/CHI PEM indicate DNA-loaded LPX, and also LPX transfection functionality after PEM embedding was maintained.^17,16^ Further, HA has a lower charge density than DNA^36,37^ and consequently should be unable to disintegrate the complex of the cationic lipid bilayers with DNA. We already demonstrated that chondroitin sulfate, a polysaccharide with higher charge density compared to HA,^34^ do not disintegrate OH4/DOPE N/P 4 LPX.^35^ Only heparin can disintegrate OH4/DOPE N/P4 LPX due to the extraordinarily high charge density. The calculated *d*_int_ values (Table 2), assuming the *d*_HH_ values of Table 1 determined via SAXS analysis are comparable for the L2-LPX phase, range between ≈12 and ≈15 Å. Such *d*_int_ values smaller than the assumed 20 Å for DNA rods^30^ were also observed in earlier research for lipid composites with high charge density indicating a very tight packing of DNA in the interlamellar space with a very low water content in the interlamellar space,^38^ a phenomenon which is also theoretically postulated by curvature fluctuations in L_α_^c^ structures.^39,40^

This brings us to the most likely hypothesis to explain the appearance of the L2-LPX phase: a decreased *d*_int_ caused by a dehydration of the DNA/water layer between the lipid bilayers. But which phenomenon can explain this loss of water in the structure?

In earlier research we showed that HA/CHI PEMs terminated with HA (sequence [HA, CHI]_5_HA was used) provide a negative surface zeta potential at pH > 3,^16^ a prerequisite for effective deposition of OH4/DOPE N/P 4 LPX with a positive zeta potential. In this preliminary work, we also postulated a deformation of PEM-deposited LPX, as indicated by a discrepancy between the LPX layer thickness inside the PEM structure determined via ellipsometry and the OH4/DOPE N/P 4 LPX size in solution.^16^ In the present work, we also examine the embedding of OH4/DOPE N/P 4 LPX in HA/CHI PEMs. The finding of the reduced *d* value of the L2-LPX supports the hypothesis of LPX deformation. The question arises which forces induce the effect? Is it based on electrostatic effects and mechanical forces, or does the difference in the physicochemical environment in PEMs result in the observed effects? Hence it was demonstrated that the dielectric properties and polarity of the environment in a PEM can be different from the bulk.^41–44^ LPX in contact with the PEMs can be influenced by local changes in dielectric properties since the LPX assembly involves an interplay of electrostatic interactions between lipid and DNA and the entropy gain resulting from counterion release after lipid/DNA complexation.^32^ Electrostatic-driven multilayer deposition and the adsorption of LPX on the PEM with terminal HA coating are based on ion-pairing interactions causing ion-exchange-type rearrangements, where water molecules are involved and counterions can be released.^45^ This can affect the hydration of the interbilayer space of LPX. According to the literature, interaction of charged PEMs with charged lipid bilayers can cause dehydration in the lipid headgroup region.^46^ Such an effect may thus also play a role in the inner LPX structure and explain the observed L2-LPX structure. It has also been postulated that PEMs can exert osmotic stress to liposomes adsorbed to PEMs,^47^ which is another possible explanation for the interlamellar dehydration observed in the present study. For cubosomes, nanoparticles with liquid crystalline inner structure, the embedding into PEMs was reported to cause decreased or increased lattice parameters, depending on the lipid composite.^48^ In another work on cubosomes encapsulated in PEMs, a decreased lattice parameter was described and attributed to the interaction with PEM.^49^ Although effects of locally changed ionic strengths can explain the reduction of interlamellar water, one also has to consider changes in the degree of protonation of the lipid composites. An increase in the protonation degree of the ionizable lipid would result in a higher charge density of the lipid bilayers, resulting in a tighter DNA complexation causing a decreased interlamellar spacing (see SAXS results described above), which has been described in terms of a change in the PEM-internal p*K*_a_.^50,51^ In this light, the conversion into the L2-LPX phase observed in this work may also be understood as a consequence of the effective local pH shifts at the PEM liquid interface.

Dehydration of the LPX structure seems to be caused by the contact of LPX with the HA/CHI PEM. Furthermore, a cover layer seems not necessary for this transition. One can claim that the LPX does not need to be fully embedded to induce the conversion to the L2-LPX phase. Nevertheless, the question is unresolved, whether a certain LPX fraction after PEM adsorption is deeply absorbed into the PEM. It is also possible that the PEM-buffer interface has a different physicochemical environment compared to the bulk which dominates the LPX environment and explains the appearance of L2-LPX. Hence this effect seems to be saturated, indicated by the coexistence of the dehydrated L2-LPX and the more strongly hydrated L1-LPX species (Tables 2 and 3). We assume that the fraction of embedded LPX in direct close contact with the PEM forms the L2-LPX phase, while a second fraction of LPX is prevented from contact with the PEM components due to interactions with other LPX or the bulk and forms the L1-LPX phase (Figure 6).

**Figure 6:**
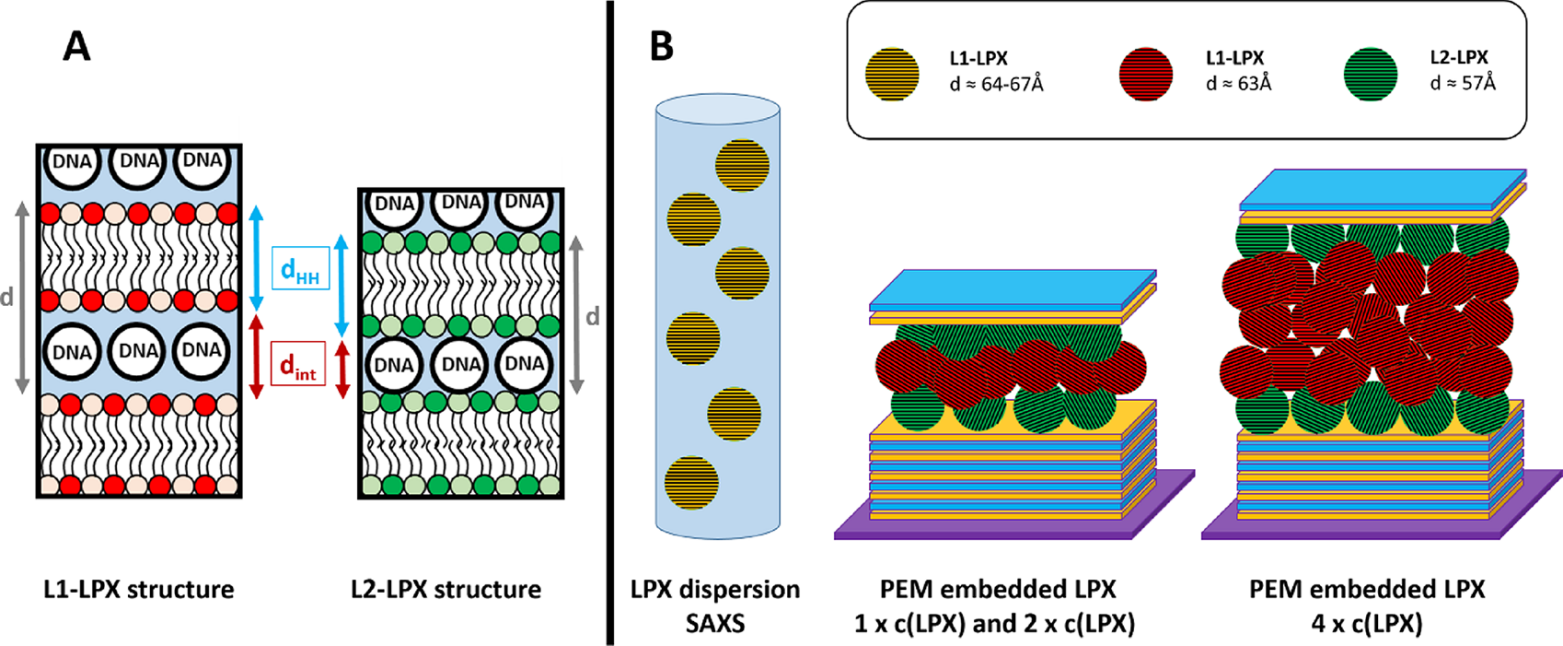
(A) Schematic illustration of the proposed differences of the two lamellar LPX structures. (B) Schematic illustration of the proposed localization of the different lamellar LPX structures in the samples.

The dehydration of LPX can be of biological relevance. For RNA-loaded LNPs it is proposed that the internal water content is connected with RNA degradation and consequently storage instabilities.^52,53^ In previous work we also could demonstrate stabilizing effects of pluronics, polyethylene glycol-polypropylene glycol-polyethylene glycol block polymers on siRNA loaded lipofectamine RNAiMAX nanoparticles.^54^ It may be possible that also here, osmotic dehydration effects improved storage stability. We hypothesize that the increased L2-LPX fraction is correlated to higher storage stability. Nevertheless, this hypothesis needs to be investigated in the future. Currently we do not know whether the conversion of LPX into the L2-LPX phase is connected with an increase or a decrease in the DNA transfer efficacy. From literature it is known that the transfection efficiency as a function of the lipid bilayer charge density in LPX is maximal at a charge density range between 0.2 and 0.35 C/m^2^.^55^ The decrease in the *d*-value observed for the conversion into the L2-LPX phase indicates an increase in the lipid bilayer charge density in L2-LPX compared to L1-LPX, but we do not know if the charge density shift for the L1-to-L2 conversion is in the increasing or decreasing part of the transfection efficiency optimum. Hence, transfection experiments with single LPX species, only L1-LPX or L2-LPX, are needed. Although our results demonstrate that adsorption time and the LPX concentration in the LPX-loading solution determine the L1/L2 ratio, we still have to learn how to control the L2-LPX phase formation in a robust way to answer this question.

## 4 Conclusion

In this work, we performed structural investigations of LPX, which were embedded in polyelectrolyte multilayers, via GISAXS. The technique allows to determine the effect of LPX immobilization in PEM on the internal structure of these nucleic acid/lipid nanoparticles. The studies show that the interaction of LPX with HA/CHI PEM is associated with changes in the repeat distance of the lamellar mesoscopic substructure of LPX. We propose that the interaction of lamellar LPX with PEMs result in a dehydrating effect on the lipid nanoparticles (reduced hydration of the interlamellar space), by observation of a L2-LPX phase with decreased *d* values compared to LPX in suspension. This L2-LPX phase occurs only upon LPX-PEM interaction. The results demonstrate that the L2-LPX fraction on PEMs coexists with a second LPX fraction, the L1-LPX phase. L1-LPX show *d* values comparable to LPX in suspension. The concentration of the LPX-loading dispersion and the incubation time determine the amount of PEM-deposited LPX as well as the L1/L2 ratio of LPX deposited onto the PEM.

## Supplementary Material



## Notes

Additional information on SAXS sample preparation, additional SAXS data, GISAXS data at 3 different angles of incidence, data on stored samples, position scans of the different GISAXS samples, gel for quantification of the DNA loading, the evaluation of peak intensity as potential tool for quantification of the DNA loading, and XRR measurements can be found in the Supporting Information (PDF)
