# Supplementary material for: Intrinsic Structure of Lipoplexes Embedded in Polyelectrolyte Multilayers
Source: Langmuir. 2026 Feb 13;42(7):5485–96. doi: 10.1021/acs.langmuir.5c05543 (PMC12937093; doi:10.1021/acs.langmuir.5c05543)
Supplement: Supplementary file 1 [file la5c05543_si_001.pdf]

Supporting Information

Intrinsic structure of lipoplexes embedded in polyelectrolyte multilayers

Maria Krabbes[a], Vincent Kampik[a], Mathilde Büttner[b], Leonard Kaysser[b], Emanuel Schneck[c], Chen Shen[d], Christian Wölk[a]*

a Institute of Pharmacy, Faculty of Medicine, Leipzig University, Eilenburger Strasse 15a, 04317 Leipzig, Germany

b Institute for Drug Discovery, Faculty of Medicine, Leipzig University, Brüderstraße 34, 04103 Leipzig, Germany

c Institute for Condensed Matter Physics, Technische Universität Darmstadt, Hochschulstrasse 8, 64289 Darmstadt, Germany

d Deutsches Elektronen-Synchrotron DESY, Notkestrasse 85, 22607 Hamburg, Germany

corresponding author: christian.woelk@medizin.uni-leipzig.de

Content:

1. SAXS sample preparation
2. Additional SAXS data
3. Samples of GISAXS at the 3 different angles of incidence
4. Stored samples
5. Position scans of the different GISAXS samples
6. Gel for quantification of the DNA loading
7. Evaluation of peak intensity as potential tool for quantification of the DNA loading
8. XRR measurements

1. SAXS sample preparation

For SAXS measurements of LPXs dispersions, three different sample preparations were performed (named sample 1 to 3). The liposome and LPX protocols had to be adapted to achieve LPX preparations of concentrations sufficient for SAXS measurements. The dry lipid films for liposome preparation were prepared from lipid stocks of 10 mg/mL. Liposomes for LPX sample 1 and 3 were prepared by adding 100 mM acetate buffer pH 5.5 to the lipid film to yield a 10.6 mg/mL lipid dispersion which was treated by 10 min sonication at 50°C. To prepare LPX sample 1, 200 µL liposome dispersion (10.6 mg/mL) was added to 330 µL pDNA solution (1 mg/mL in solvent from supplier) in one step and incubated for 20 min at 22°C and 120 rpm for LPX formation. The turbid LPX dispersion was centrifuged for 30 min at 20 °C and 30,000 g. The supernatant was removed and the LPX pellet re-suspended in 50 µL 100 mM acetate buffer pH 5.5 to yield a final LPX concentration of 6.6 mg/mL (calculated for DNA content). To prepare LPX sample 3, 50 µL pDNA solution (6.6 mg/mL in PCR grade water) were added to 200 µL liposome dispersion (10.6 mg/mL) in one step and incubated for 20 min at 22°C and 120 rpm for LPX formation. The turbid LPX dispersion had a final concentration of 1.32 mg/mL (calculated for DNA content). For the preparation of the LPX sample 2, A dried lipid film of 2.1 mg total lipid was dispersed in 50 µL 100 mM acetate buffer pH 5.5 (10 min sonication at 50°C). 50 µL pDNA solution (6.6 mg/mL in PCR grade water) were added to the lipid dispersion followed by incubated for 20 min at 22°C and 120 rpm. The LPX concentration was 3.3 mg/mL (calculated for DNA content).

2. Additional SAXS data

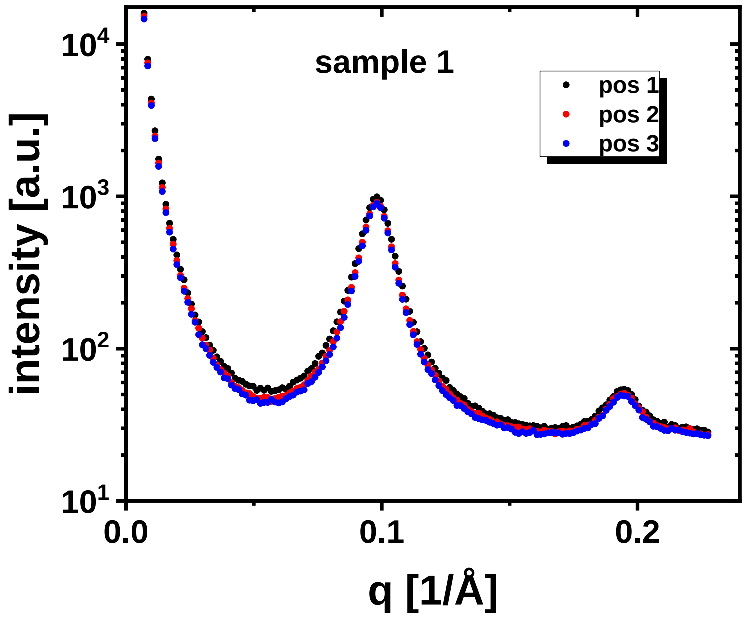

Figure S1: SAXS pattern of OH4/DOPE NP4 LPX sample 1 in acetate buffer pH 5.5 without baseline correction at 3 different positions of the capillary.

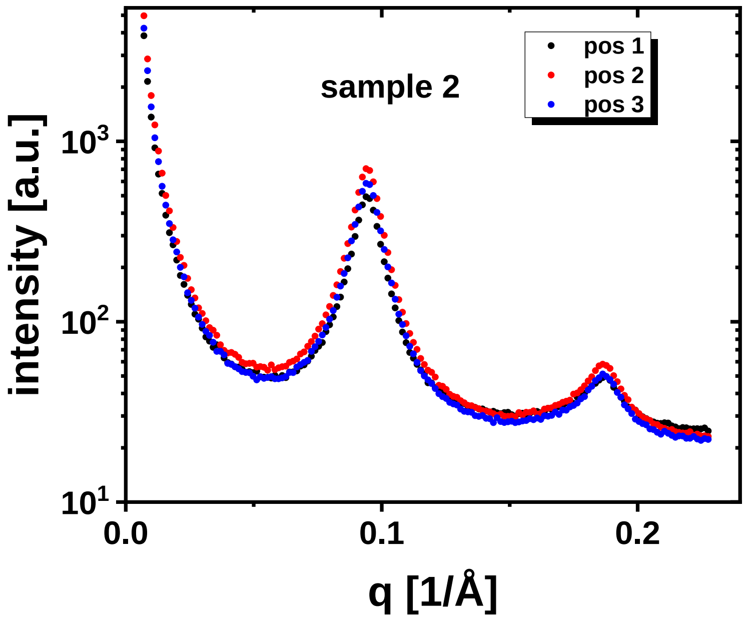

Figure S2: SAXS pattern of OH4/DOPE NP4 LPX sample 2 in acetate buffer pH 5.5 without baseline correction at 3 different positions of the capillary.

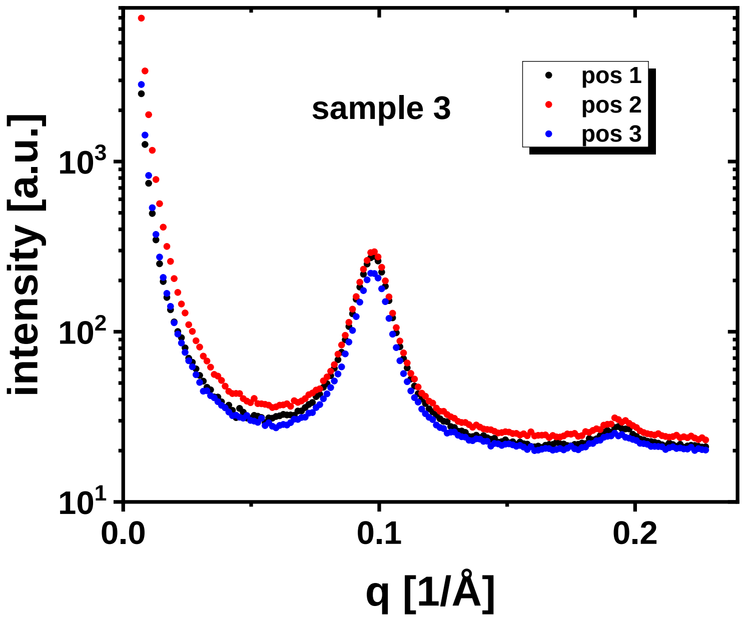

Figure S3: SAXS pattern of OH4/DOPE NP4 LPX sample 3 in acetate buffer pH 5.5 without baseline correction at 3 different positions of the capillary.

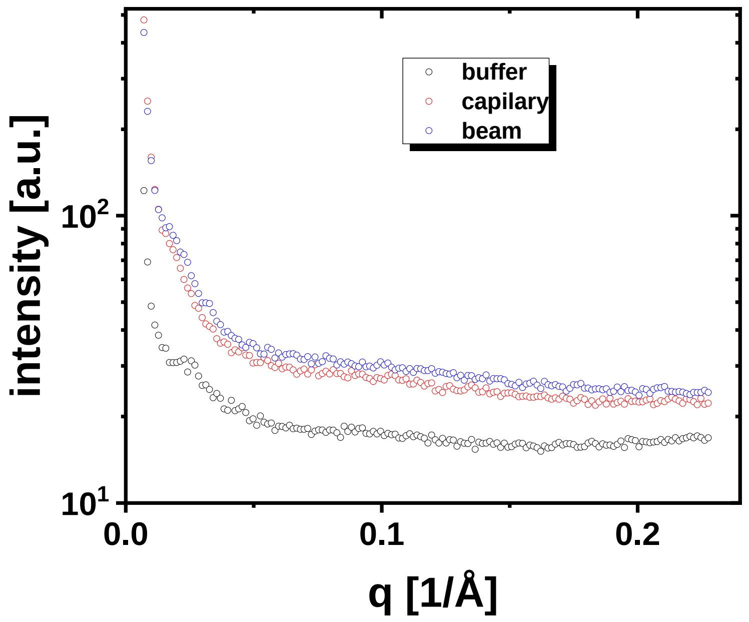

Figure S4: SAXS pattern background of the capillary filled with buffer (buffer), the empty capillary (capillary) and the empty beam path (beam).

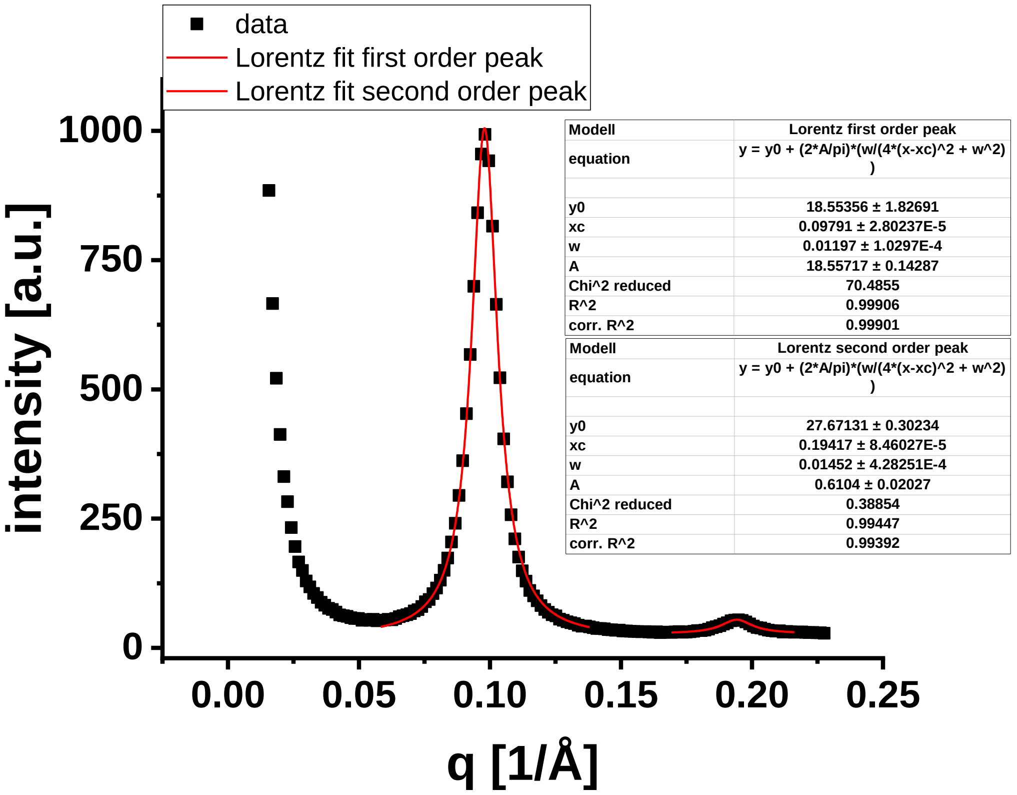

Figure S5: Lorentz fit of SAXS pattern of OH4/DOPE NP4 LPX sample 1 in acetate buffer pH 5.5 separately fitted for the single peaks at q_1 and q_2.

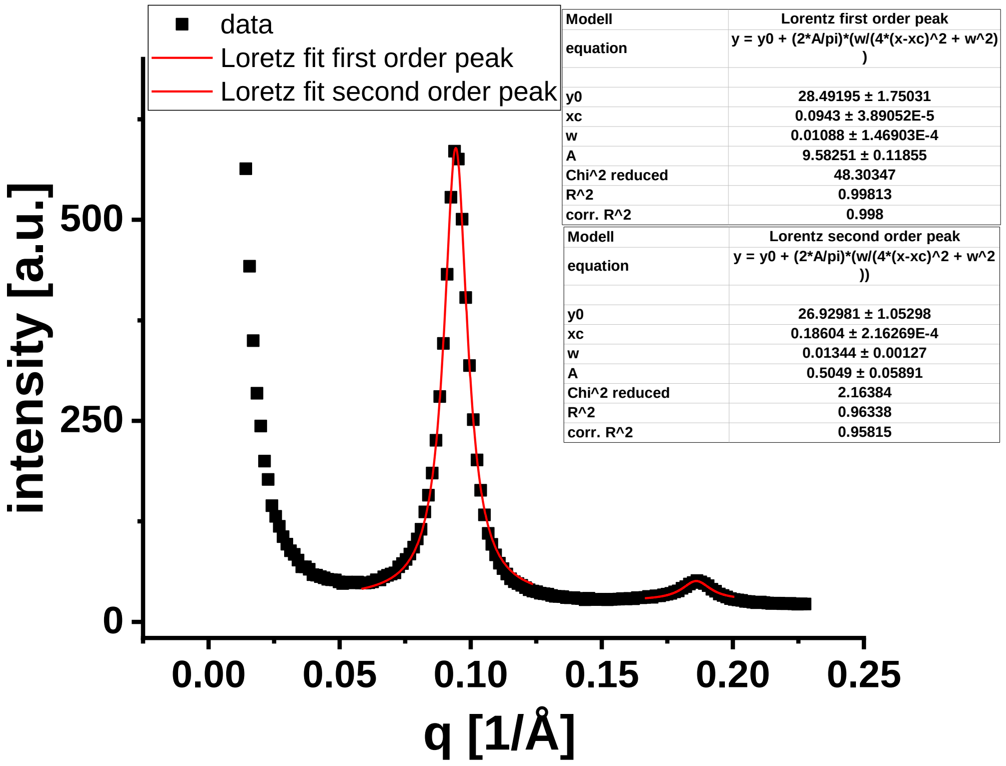

Figure S6: Lorentz fit of SAXS pattern of OH4/DOPE NP4 LPX sample 2 in acetate buffer pH 5.5 separately fitted for the single peaks at q_1 and q_2.

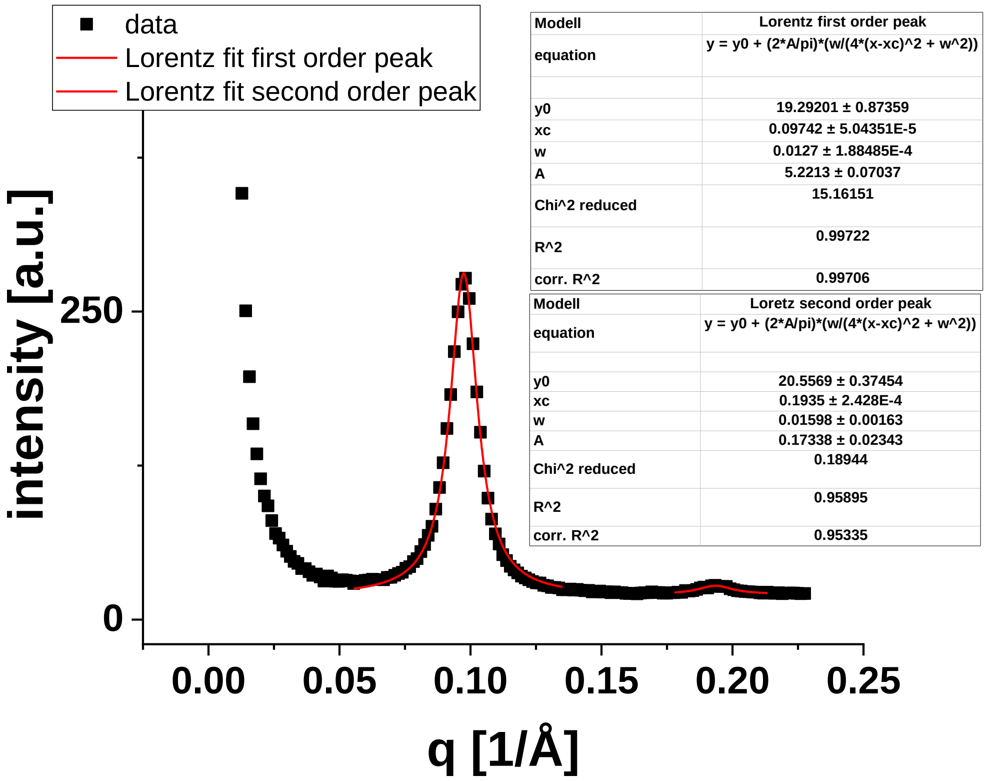

Figure S7: Lorentz fit of SAXS pattern of OH4/DOPE NP4 LPX sample 3 in acetate buffer pH 5.5 separately fitted for the single peaks at q_1 and q_2..

Table S1: peak maxima (q_n) of the Lorentz fit of measured SAXS curves and the *d* values calculated from the first order peak

**Measurement position**	**sample 1**	**sample 2**	**sample 3**
first order peak (n=1)			
	q_n [1/Å]	q_n [1/Å]	q_n [1/Å]
**position 1**	0.09791 ± 0.00002	0.09425 ± 0.00004	0.09742 ± 0.00005
**position 2**	0.09794 ± 0.00003	0.09427 ± 0.00004	0.09744 ± 0.00005
**position 3**	0.09797 ± 0.00002	0.09430 ± 0.00004	0.09739 ± 0.00005
second order peak (n=2)			
	q_n [1/Å]	q_n [1/Å]	q_n [1/Å]
**position 1**	0.1942 ± 0.0001	0.1855 ± 0.0003	0.1930 ± 0.0002
**position 2**	0.1943 ± 0.0001	0.1858 ± 0.0003	0.1934 ± 0.0003
**position 3**	0.1942 ± 0.0001	0.1860 ± 0.0002	0.1930 ± 0.0003
repeat distance			
	d [Å]	*d* [Å]	*d* [Å]
**position 1**	64.17 ± 0.01	66.67 ± 0.03	64.50 ± 0.03
**position 2**	64.15 ±0.02	66.65 ± 0.03	64.48 ± 0.03
**position 3**	64.13 ± 0.02	66.63 ± 0.03	64.52 ± 0.03

Electron Density Profile obtained from SAXS data

Low resolution electron density contrast $\Delta\rho_e$ of the LPX in dispersion, calculated from three orders of Bragg peaks. In all plots, the full *z* scale represents the distance from the mid-plane of the interbilayer space to the mid-plane of the next interbilayer space, within which one bilayer is located at. The two high electron density regions near z ~ +/- 20Å are the two headgroup region of a bilayer. The curves from the lowest to the highest headgroup electron density contrast (arb. u.) correspond to the assumed F_3 from the lowest to the highest value (see the methods section). The negative electron density contrast region in the middle corresponds to the hydrocarbon chain region. Note that the increased $\Delta\rho_e$ in this region is an artefact due to the lack of the form factor value at the 4th order Bragg peak position, and this has no effect to the determination of head-to-head distance d_{HH}.

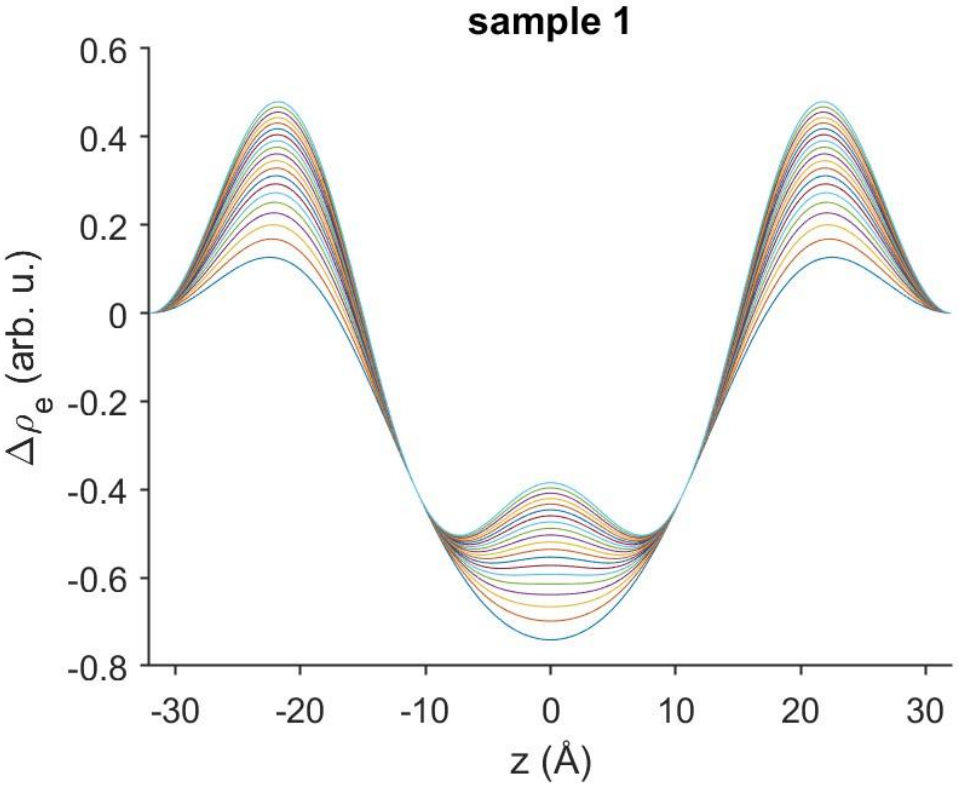

Figure S8: qualitative Fourier reconstruction to yield the head-to-head thickness d_{HH} of the bilayers of SAXS sample 1.

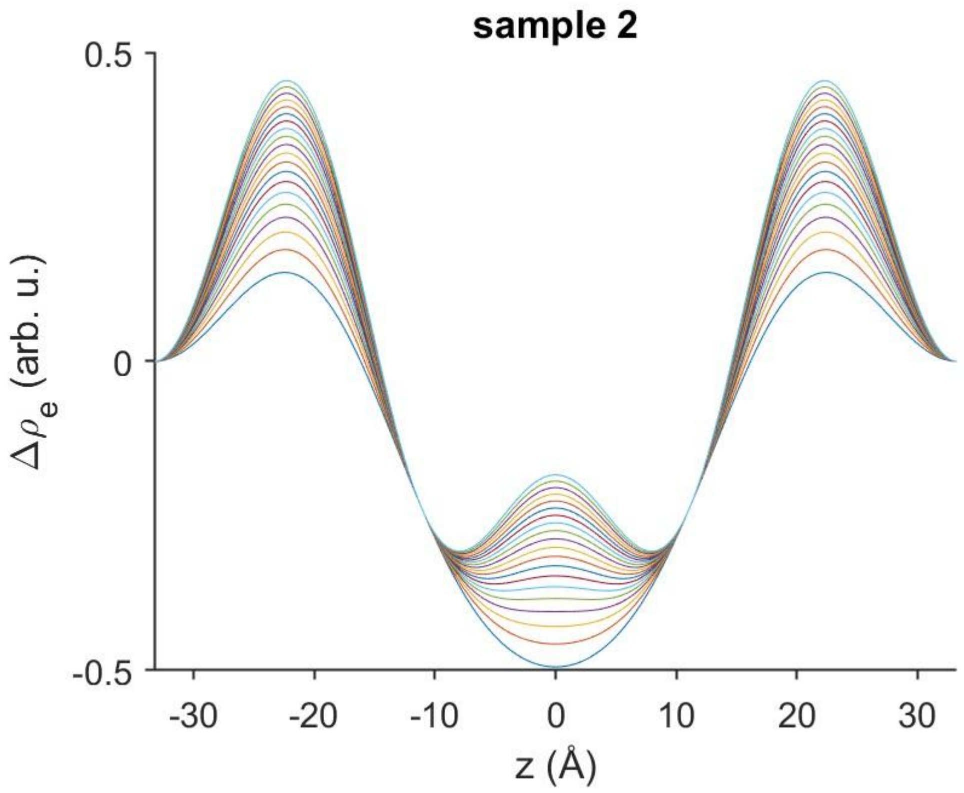

Figure S9: qualitative Fourier reconstruction to yield the head-to-head thickness d_{HH} of the bilayers of SAXS sample 2.

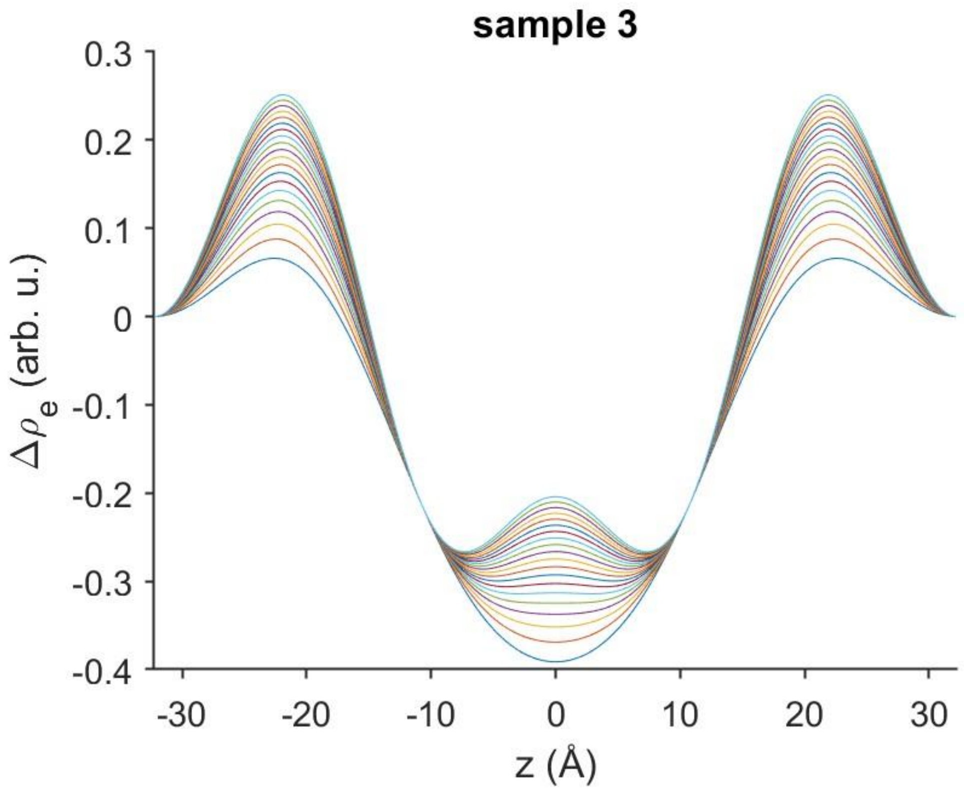

Figure S10: qualitative Fourier reconstruction to yield the head-to-head thickness d_{HH} of the bilayers of SAXS sample 2.

3. Samples of GISAXS at the 3 different angles of incidence

A clear Scherer rings were observed in the GISAXS signal. Nevertheless, in some samples a ”ghost ring” occurs from the same Bragg reflection of the deposited layer under the total reflection of the incident beam from the substrate, when the incident angle is lower than the critical angle of the substrate. At 18 keV, the measurement at 0.07° incidence meets this condition, as the critical angle of the air-silicon interface is about 0.095°. The dominant Bragg reflection occurs when the incident beam is directly diffracted by the lamellar structure in the deposited layer, exhibiting the strongest Scherrer ring. A secondary Bragg reflection occurs when the reflected beam from the silicon substrate surface re-enters the deposited layer and gets diffracted, exhibiting a weaker “ghost ring”. The ghost ring is weaker because the reflected beam from the wafer surface has been attenuated by the deposited layer once. Since the primary beams of the two diffraction events are the incident beam and the reflected beam from the substrate, their orientation differs in the vertical direction by two times the incident angle. Accordingly, the main diffraction ring and the ghost ring are offset vertically by two times the incident angle. At higher incident angles where the reflection from the wafer surface is much weaker, the ghost ring is negligible.

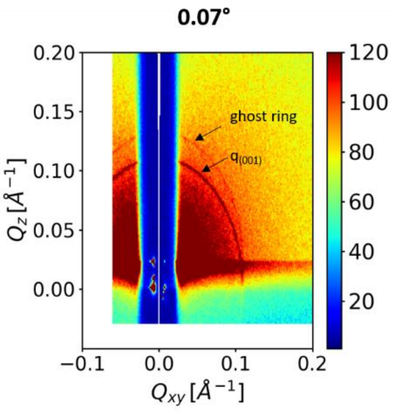

Figure S11: Detector image of the GISAXS signal of the coating sequence PEM-LPX-HA-CHI at different incidence angles.

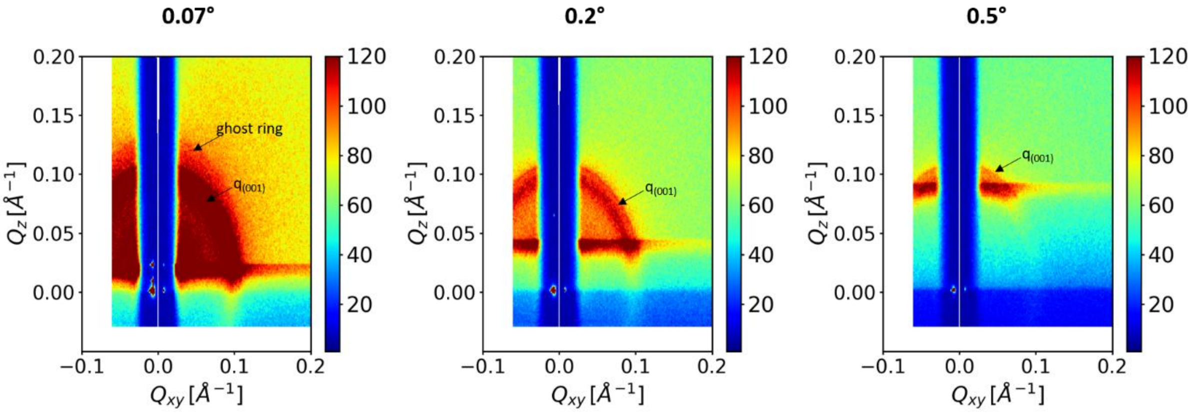

Figure S12: Detector image of the GISAXS signal of the coating sequence PEM-LPX-HA-CHI 4 x c(LPX) at different incidence angles.

4. Stored samples

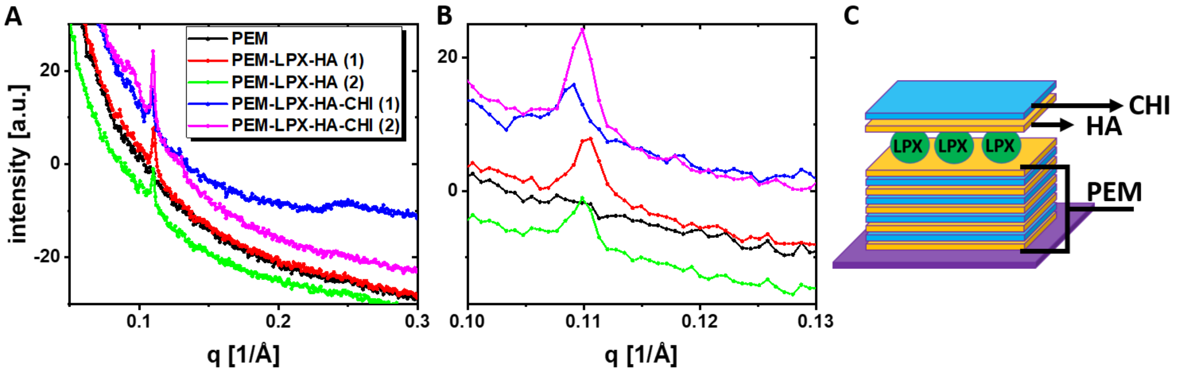

Figure S13: GISAXS experiments of stored samples at different processing levels. A) 1D GISAXS diffractogram of stored PEMs at different processing steps (depicted in C). (1) and (2) label two independent samples with the same structure. GISAXS diffractogram of the samples at 4 different positions are shown in Figure S14-17) to demonstrate homogeneity of the samples. B) Detailed q range from A.

Table S2 LPX Bragg peak position q_1 and calculated d value of the L2 LPX phase obtained from GISAXS measurement of OH4/DOPE NP4 LPX on or embedded in PEMs as mean ± standard deviation of 4 different positions. The samples were stored for 4-5 days in buffer before measurement. The samples were prepared with a LPX incubation solution of 4.33 µg/mL (calculated for DNA content).

sample name	q_1 [1/Å]	d [Å]	coverlayer on top of LPX
**PEM-LPX-HA (1)**	0.1101 ± 0.0002	57.04 ± 0.12	HA
**PEM-LPX-HA (2)**	0.1097 ± 0.0001	57.28 ± 0.03	HA
**PEM-LPX-HA-CHI (1)**	0.1089 ± 0.0002	57.68 ± 0.12	HA/CHI
**PEM-LPX-HA-CHI (2)**	0.1098 ± 0.0001	57.2 ± 0.04	HA/CHI

5. Position scans of the different GISAXS samples

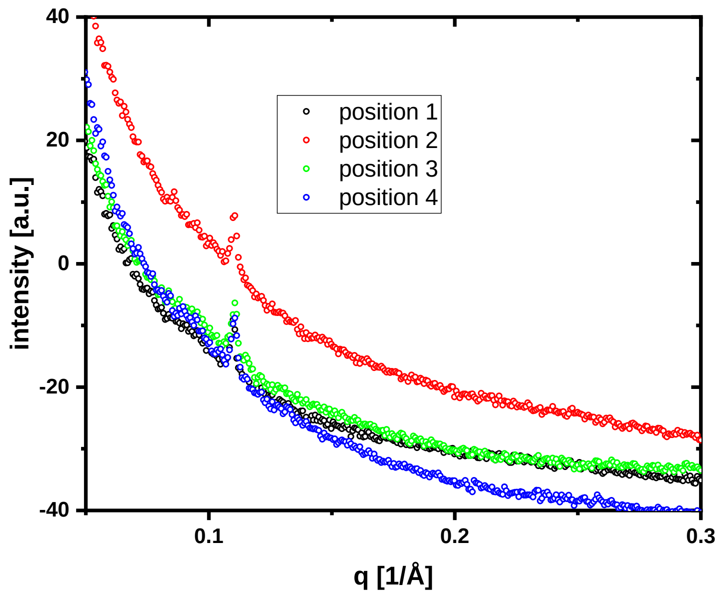

Figure S14: GISAXS diffraction patterns obtained by azimuthal integration of the 2D detector image at beam incidence of 0.07°of the stored PEM coated silica wafer with the PEM system PEM-LPX-HA (1) measured at 4 different positions.

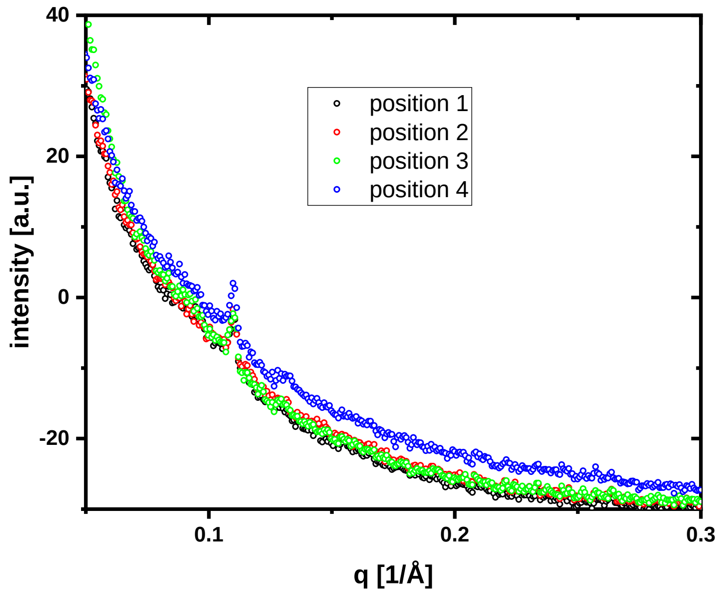

Figure S15: GISAXS diffraction patterns obtained by azimuthal integration of the 2D detector image at beam incidence of 0.07°of the stored PEM coated silica wafer with the PEM system PEM-LPX-HA (2) measured at 4 different positions.

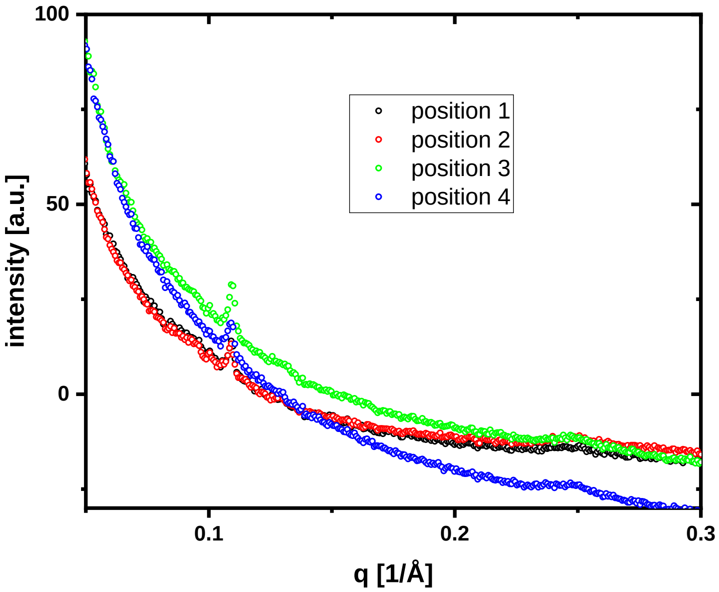

Figure S16: GISAXS diffraction patterns obtained by azimuthal integration of the 2D detector image at beam incidence of 0.07°of the stored PEM coated silica wafer with the PEM system PEM-LPX-HA-CHI (1) measured at 4 different positions.

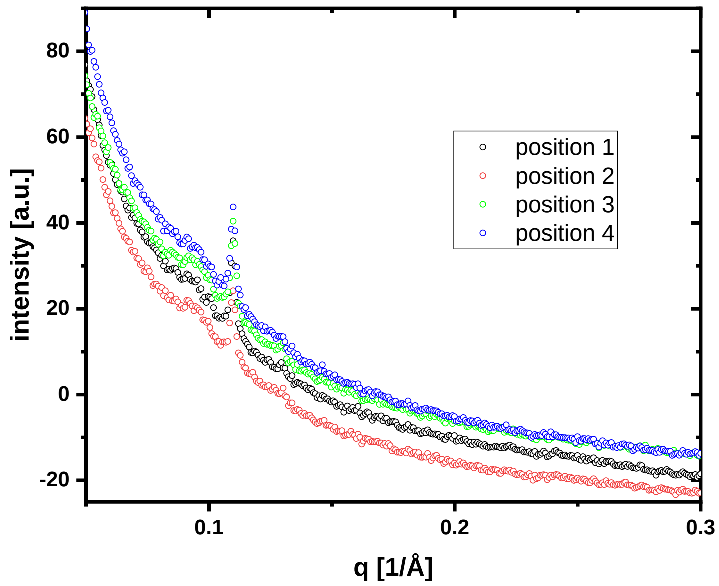

Figure S17: GISAXS diffraction patterns obtained by azimuthal integration of the 2D detector image at beam incidence of 0.07°of the stored PEM coated silica wafer with the PEM system PEM-LPX-HA-CHI (2) measured at 4 different positions.

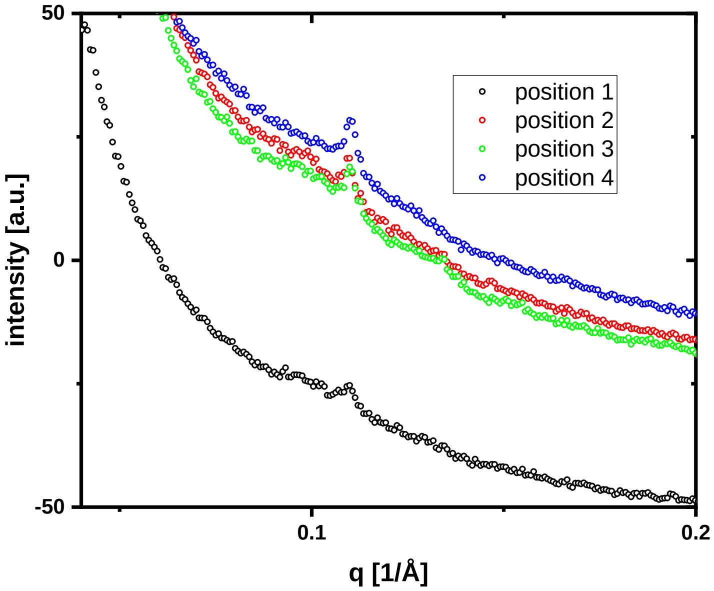

Figure S18: GISAXS diffraction patterns obtained by azimuthal integration of the 2D detector image at beam incidence of 0.07°of the silica wafer with the PEM system freshly coated with the PEM-LPX-HA-CHI coating with the 1 x c(LPX) loading quantity measured at 4 different positions.

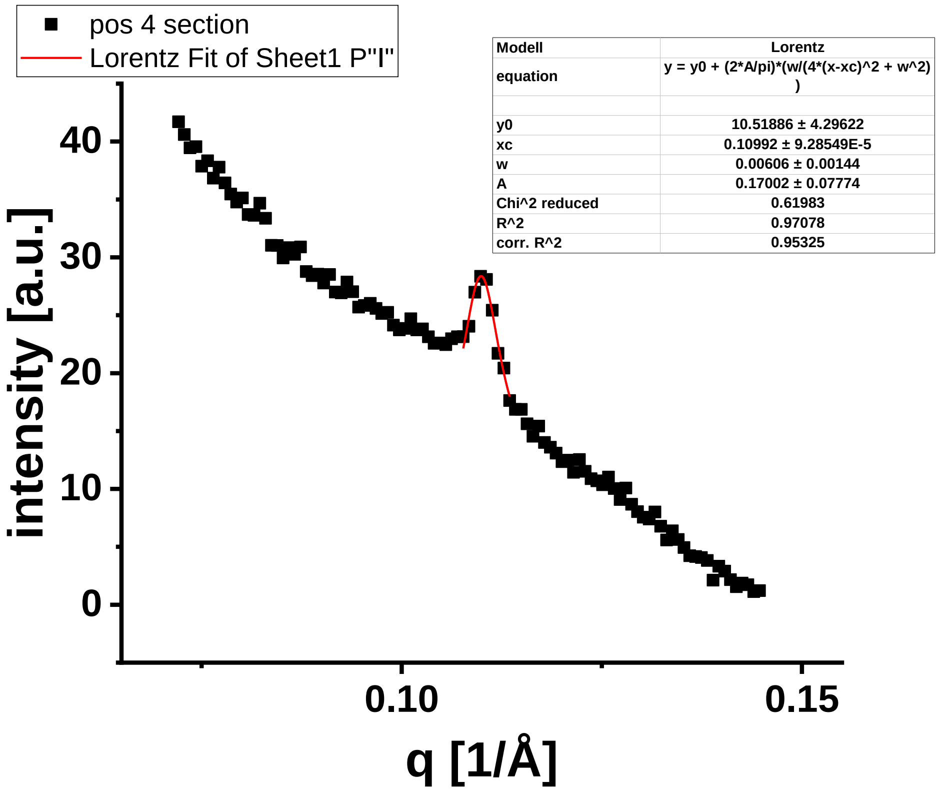

Figure S19: Lorentz fit of GISAXS diffraction pattern of PEM-LPX-HA-CHI coating with the 1 x c(LPX) loading quantity measured at position 4 (see Figure S18).

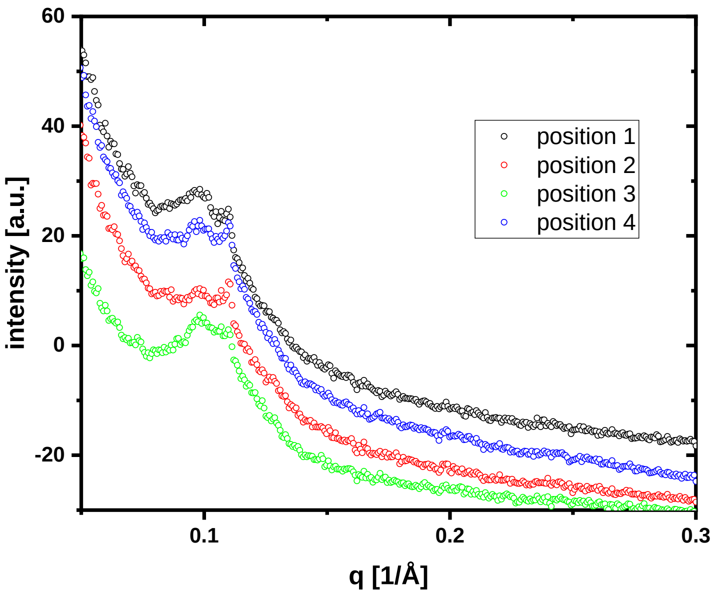

Figure S20: GISAXS diffraction patterns obtained by azimuthal integration of the 2D detector image at beam incidence of 0.07°of the silica wafer with the PEM system freshly coated with the PEM-LPX-HA-CHI coating with the 2 x c(LPX) loading quantity measured at 4 different positions.

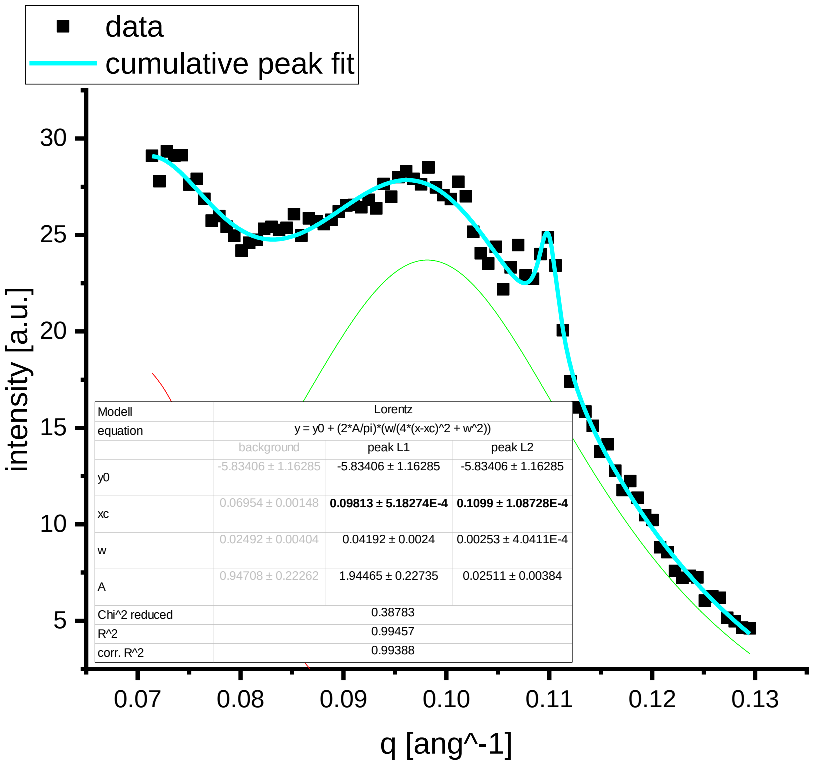

Figure S21: Lorentz fit of GISAXS diffraction pattern of PEM-LPX-HA-CHI coating with the 2 x c(LPX) loading quantity measured at position 1 (see Figure S20).

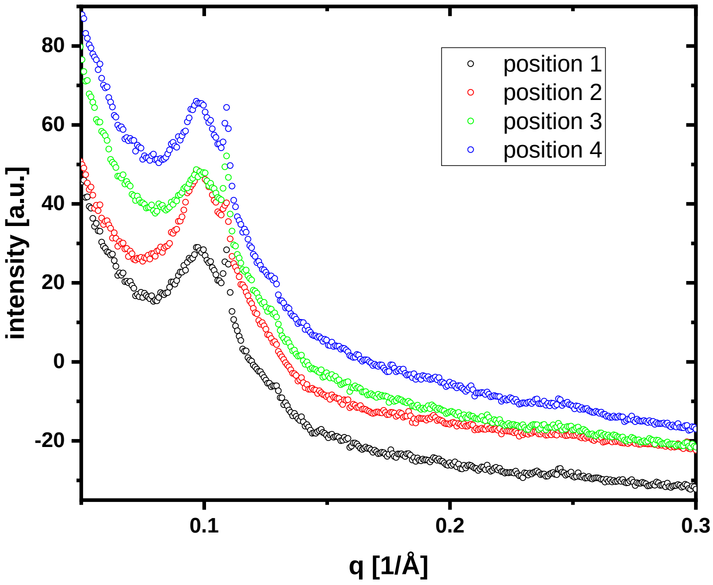

Figure S22: GISAXS diffraction patterns obtained by azimuthal integration of the 2D detector image at beam incidence of 0.07°of the silica wafer with the PEM system freshly coated with the PEM-LPX-HA-CHI coating with the 4 x c(LPX) loading quantity measured at 4 different positions.

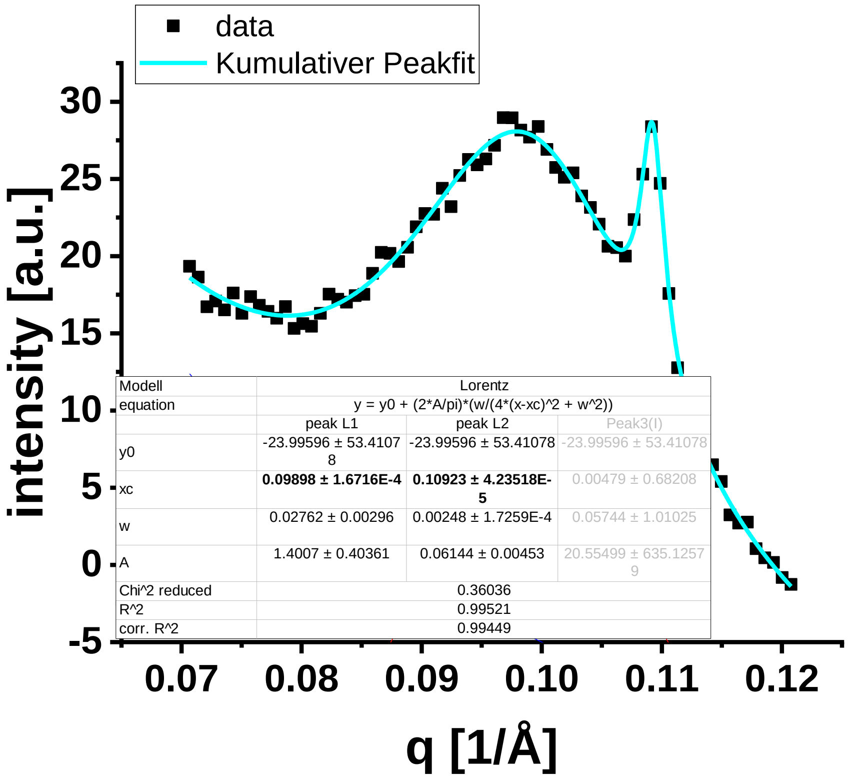

Figure S23: Lorentz fit of GISAXS diffraction pattern of PEM-LPX-HA-CHI coating with the 4 x c(LPX) loading quantity measured at position 1 (see Figure S22).

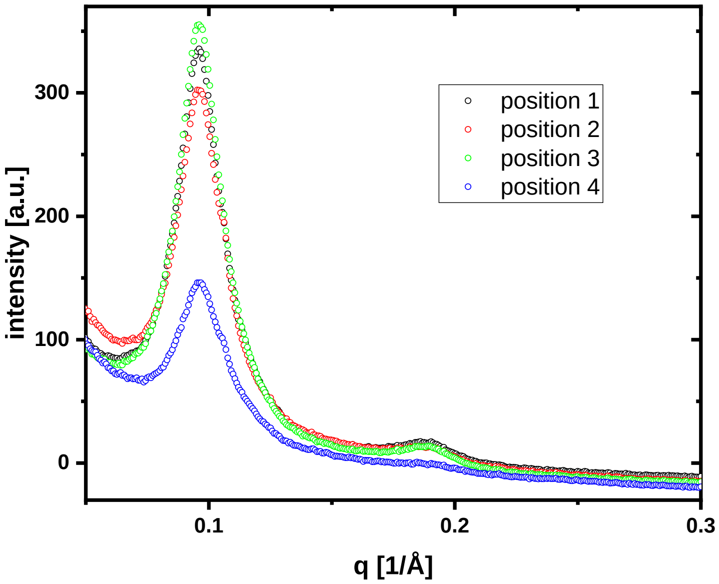

Figure S24: GISAXS diffraction patterns obtained by azimuthal integration of the 2D detector image at beam incidence of 0.07°of the silica wafer with the PEM system freshly coated with the PEM-LPX-HA-CHI coating with the 8 x c(LPX) loading quantity measured at 4 different positions.

Modell	Lorentz		
equation	y = y0 + (2*A/pi)*(w/(4*(x-xc)^2 + w^2))		
	peak L1	peak L2	Peak3(l)
y0	-11.0274 ± 203.20859	-11.0274 ± 203.20859	-11.0274 ± 203.20859
xc	**0.0964 ± 5.59308E-5**	**0.10637 ± 1.35664E-4**	-0.05742 ± 2.24677
w	0.02097 ± 5.16021E-4	0.00381 ± 6.15549E-4	0.07924 ± 5.09794
A	3.71084 ± 0.20446	0.0586 ± 0.0115	87.13314 ± 7705.29028
Chi^2 reduced	0.75388		
R^2	0.99931		
corr. R^2	0.9992		

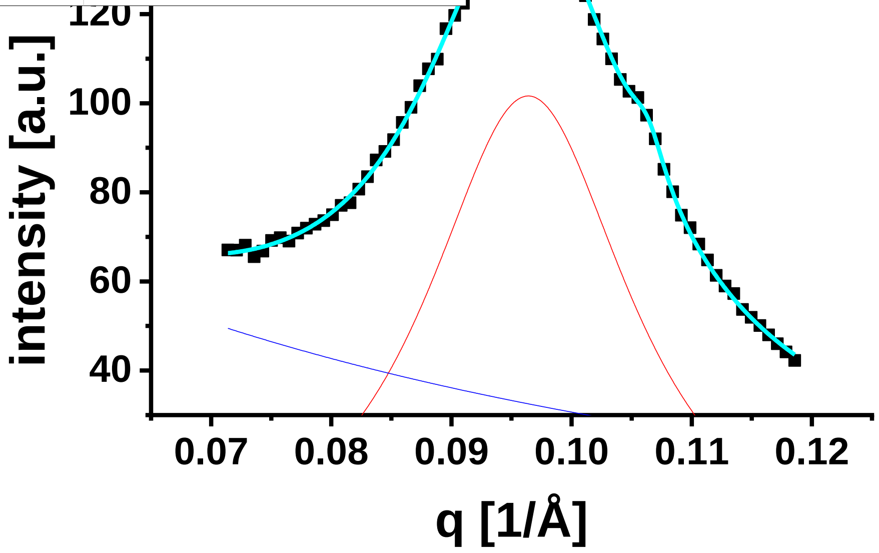

Figure S25: Lorentz fit of GISAXS diffraction pattern of PEM-LPX-HA-CHI coating with the 8 x c(LPX) loading quantity measured at position 4 (see Figure S24).

Table S3: peak maxima (q_1) of the Lorentz fit of measured SAXS curves and the *d* values calculated from the first order peak of the L1- and L2-LPX phase.

**1 x c(LPX)**	**L1 - LPX**	**L2 LPX**
	**q_1 ± fit error [1/Å]**	**q_1 ± fit error [1/Å]**
position 1	0.100 ± 0.0007	0.10958 ± 0.00003
position 2	0.099 ± 0.0004	0.10898 ± 0.00022
position 3	0.105 ± 0.0005	0.10989 ± 0.00003
position 4	0.106 ± 0.0010	0.10992 ± 0.00009
	***d* ± fit error [Å]**	***d* ± fit error [Å]**
position 1	62.83 ± 0.44	57.34 ± 0.02
position 2	63.47 ± 0.26	57.65 ± 0.12
position 3	59.84 ± 0.29	57.18 ± 0.02
position 4	59.28 ± 0.56	57.16 ± 0.05
**2 x c(LPX)**	**q_1 ± fit error [1/Å]**	**q_1 ± fit error [1/Å]**
position 1	0.0981 ± 0.0005	0.1099 ± 0.0001
position 2	0.1022 ± 0.0009	0.1099 ± 0.0000
position 3	0.1017 ± 0.0007	0.1100 ± 0.0002
position 4	0.1010 ± 0.0008	0.1101 ± 0.0001
	***d* ± fit error [Å]**	***d* ± fit error [Å]**
position 1	64.03 ± 0.34	57.17 ± 0.06
position 2	61.50 ± 0.52	57.19 ± 0.00
position 3	61.78 ± 0.44	57.11 ± 0.09
position 4	62.23 ± 0.46	57.09 ± 0.05
**4 x c(LPX)**	**q_1 ± fit error [1/Å]**	**q_1 ± fit error [1/Å]**
position 1	0.0990 ± 0.0002	0.10923 ± 0.00004
position 2	0.0990 ± 0.0001	0.10896 ± 0.00007
position 3	0.0996 ± 0.0002	0.10910 ± 0.00004
position 4	0.0990 ± 0.0002	0.10920 ± 0.00004
	***d* ± fit error [Å]**	***d* ± fit error [Å]**
position 1	63.48 ± 0.11	57.52 ± 0.02
position 2	63.45 ± 0.08	57.67 ± 0.04
position 3	63.12 ± 0.12	57.59 ± 0.02
position 4	63.44 ± 0.10	57.54 ± 0.02
**8 x c(LPX)**	**q_1 ± fit error [1/Å]**	**q_1 ± fit error [1/Å]**
position 1	0.0956 ± 0.0001	0.1061 ± 0.0004
position 2	0.0962 ± 0.00002	0.1064 ± 0.00004
position 3	0.0961 ± 0.00002	0.1062 ± 0.0002
position 4	0.0964 ± 0.0001	0.1064 ± 0.0001
	***d* ± fit error [Å]**	***d* ± fit error [Å]**
position 1	65.70 ± 0.05	59.23 ± 0.22
position 2	65.30 ± 0.01	59.05 ± 0.02
position 3	65.36 ± 0.01	59.15 ± 0.10
position 4	65.18 ± 0.04	59.07 ± 0.08

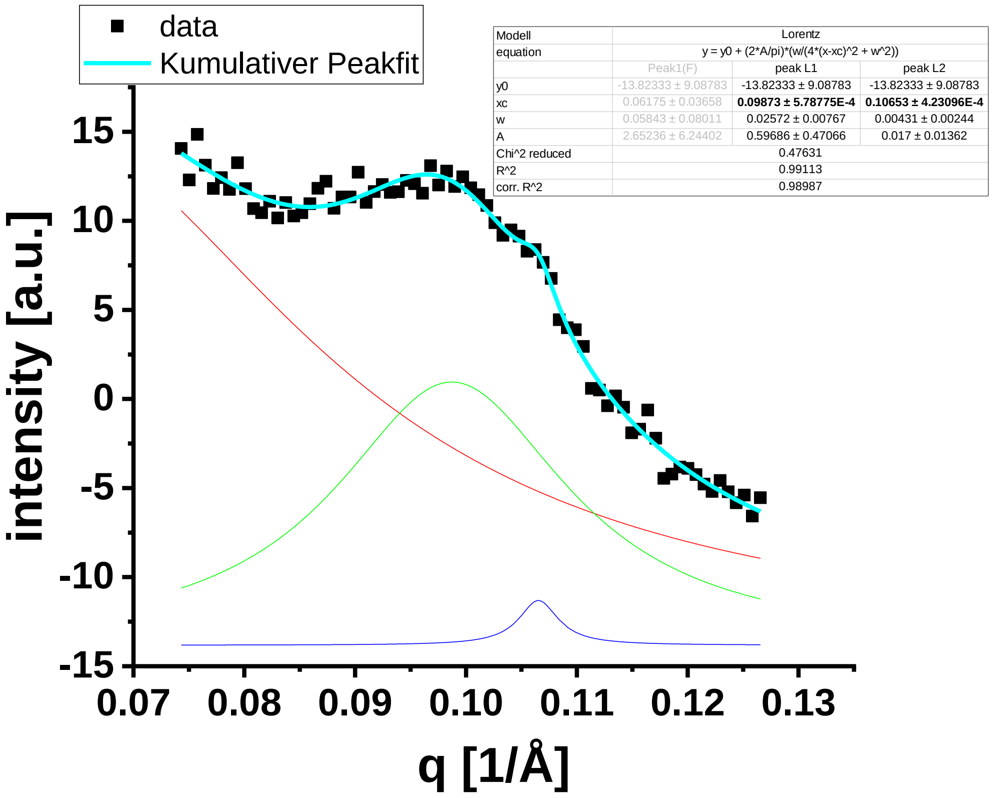

Figure S26: Lorentz fit of the GISAXS *in-situ* LPX deposition experiment, time point 45 min

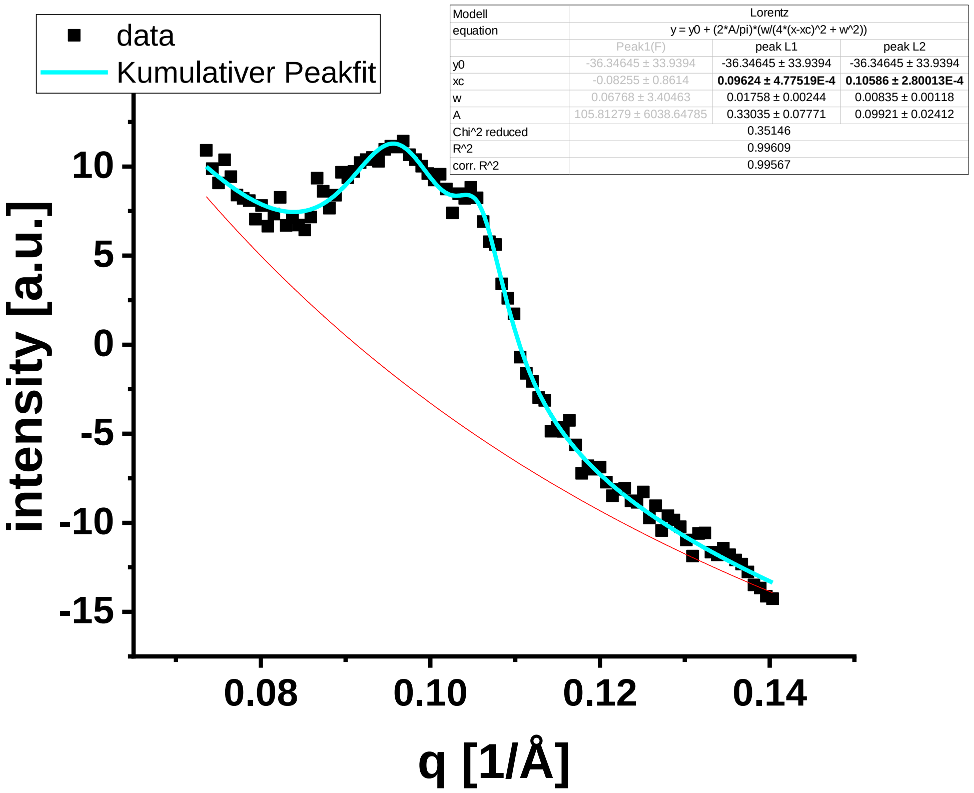

Figure S27: Lorentz fit of the GISAXS *in-situ* LPX deposition experiment, time point 60 min

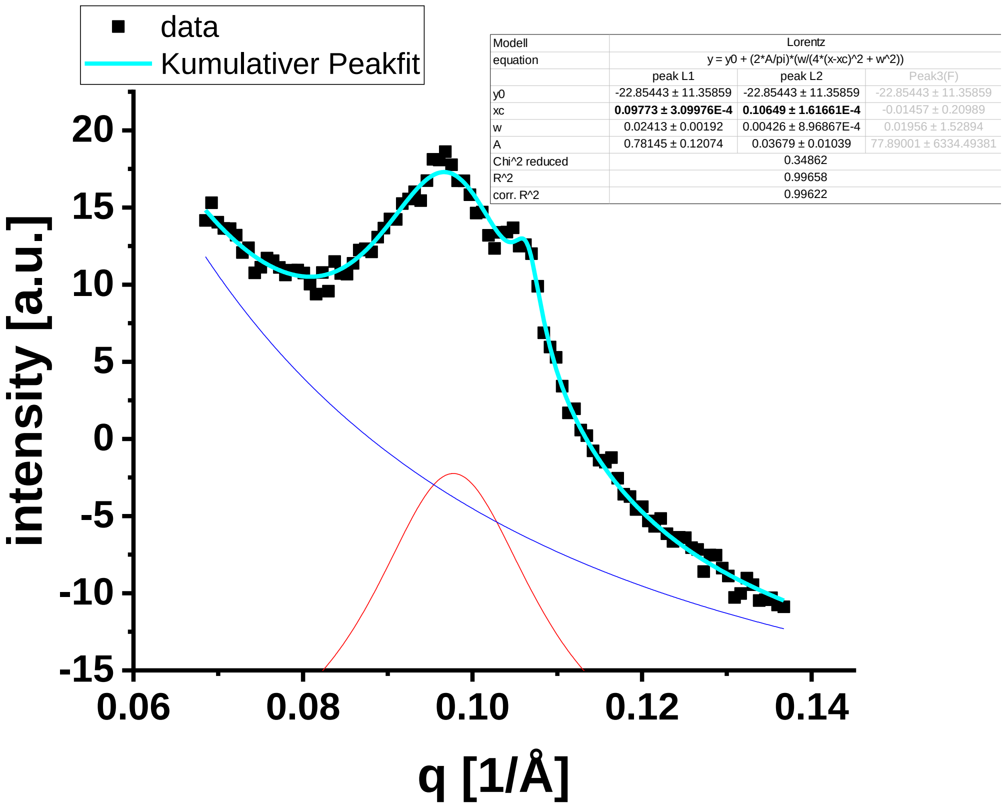

Figure S28: Lorentz fit of the GISAXS *in-situ* LPX deposition experiment, time point 90 min

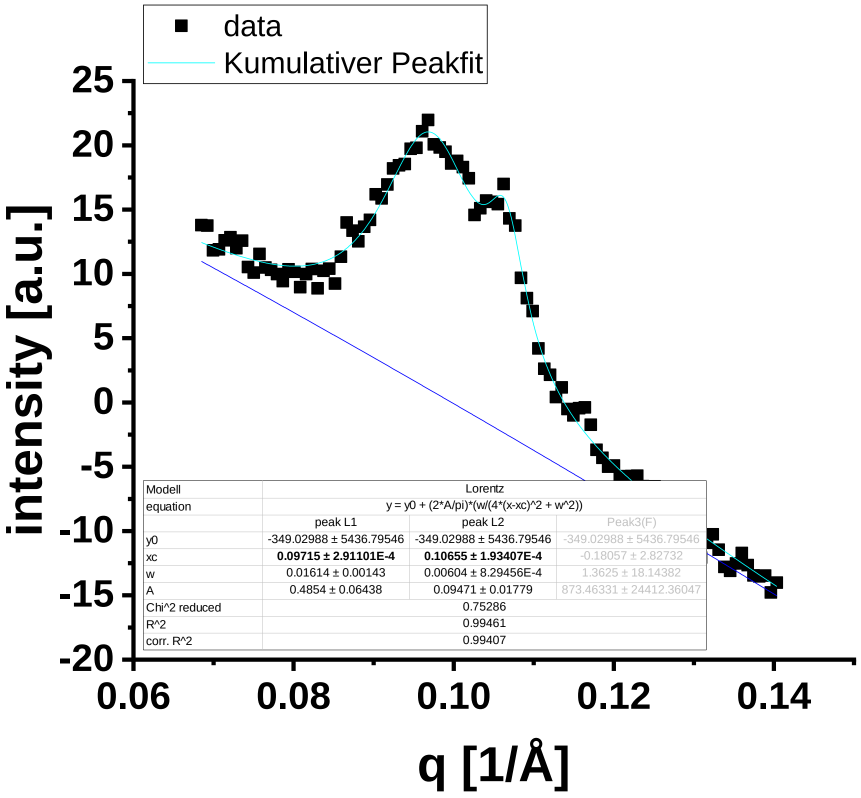

Figure S29: Lorentz fit of the GISAXS *in-situ* LPX deposition experiment, time point 120 min

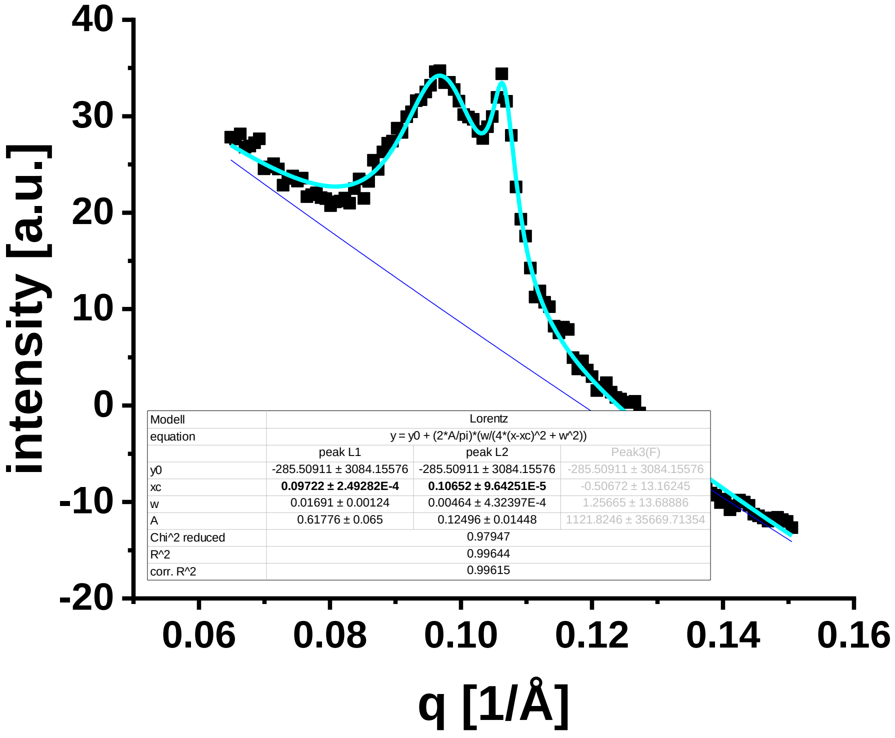

Figure S30: Lorentz fit of the GISAXS *in-situ* LPX deposition experiment, time point 180 min

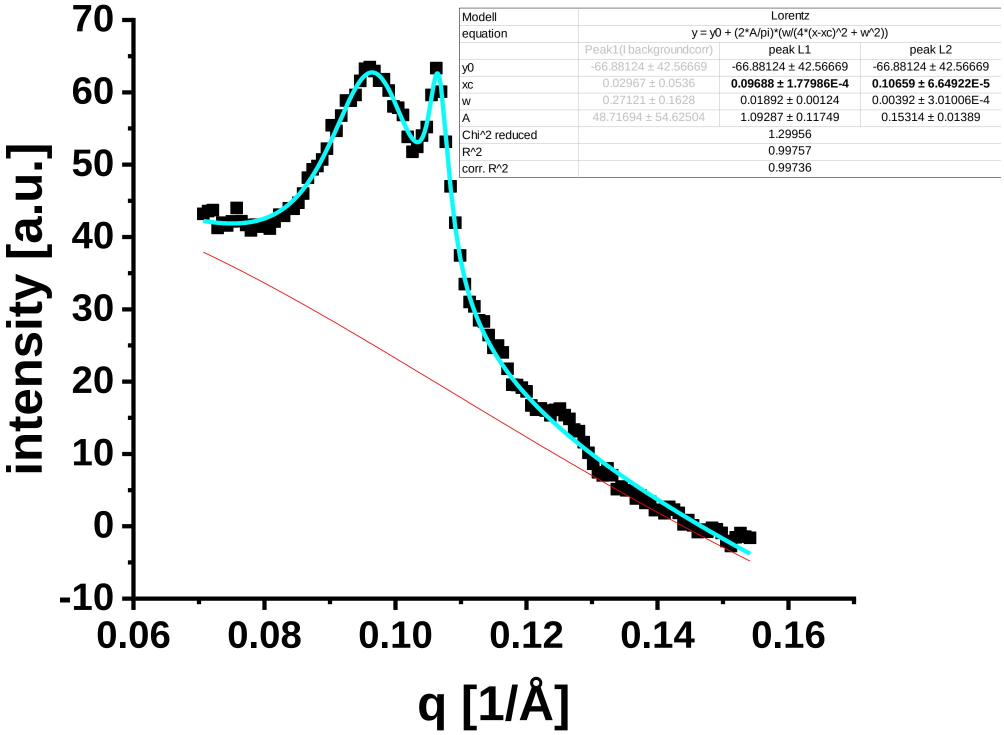

Figure S31: Lorentz fit of the GISAXS *in-situ* LPX deposition experiment, time point 240 min

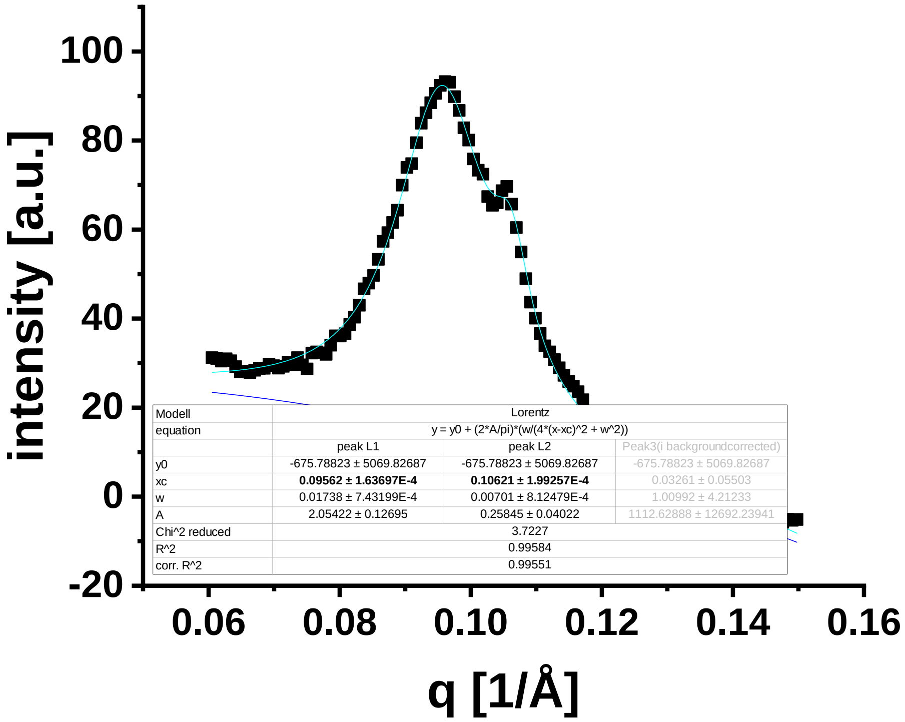

Figure S32: Lorentz fit of the GISAXS *in-situ* LPX deposition experiment, time point 540 min

6. Gel for quantification of the DNA loading

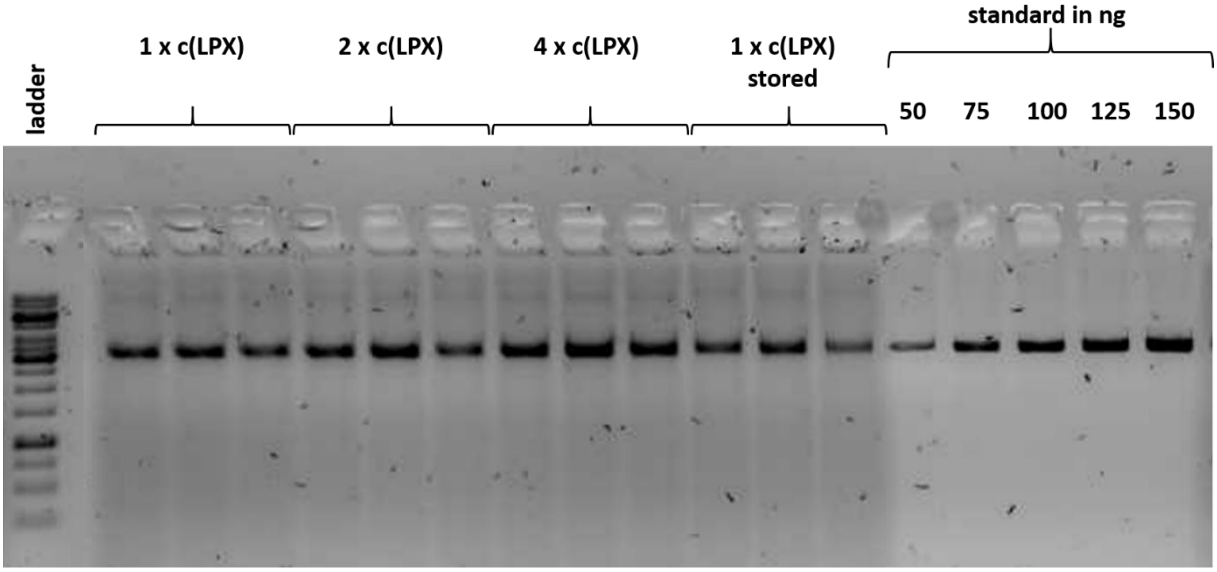

Figure S33: Gel electrophoresis of the DNA quantification procedure described in chapter 2.9 “Quantification of the DNA loading” of the main article.

7. Evaluation of peak intensity as potential tool for quantification of the DNA loading

The samples loaded with an increasing amount of LPX (samples presented in Figure 4 of the main text labelled with 1 x c(LPX), 2 x c(LPX), and 4 x c(LPX)) are evaluated regarding the peak intensity. For determination of the peak area, the diffraction curve was baseline corrected and two Lorentzian peaks were fitted to the Bragg signal using OriginPro 2019 software. Example results are given in Figures S34-S36.

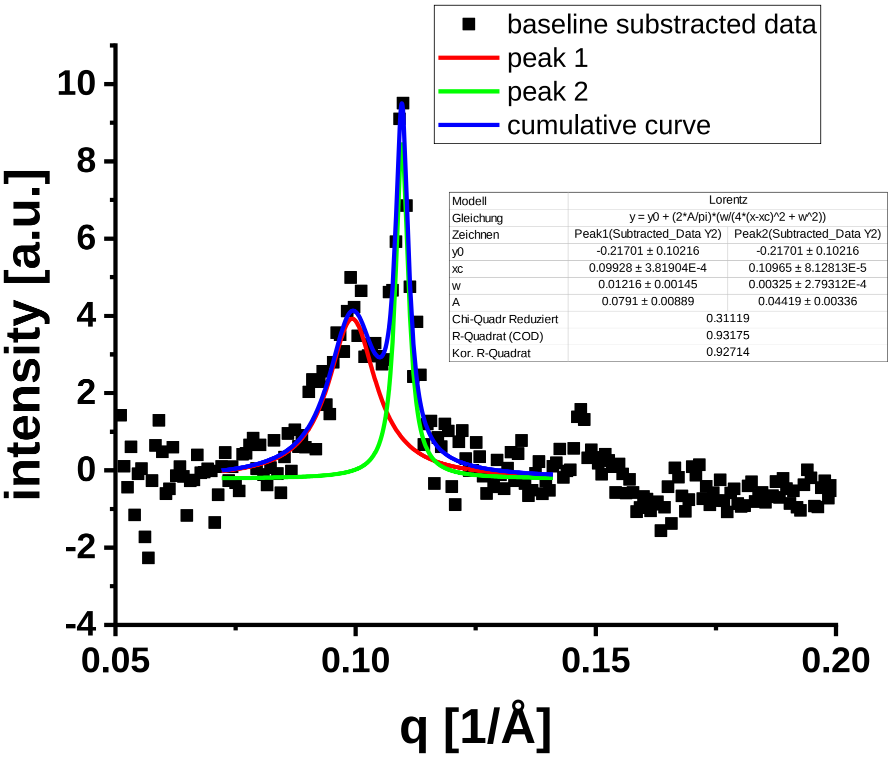

Figure S34: Multiple Lorentz fit of baseline subtracted GISAXS diffraction pattern of PEM-LPX-HA-CHI coating with the 1 x c(LPX) loading quantity.

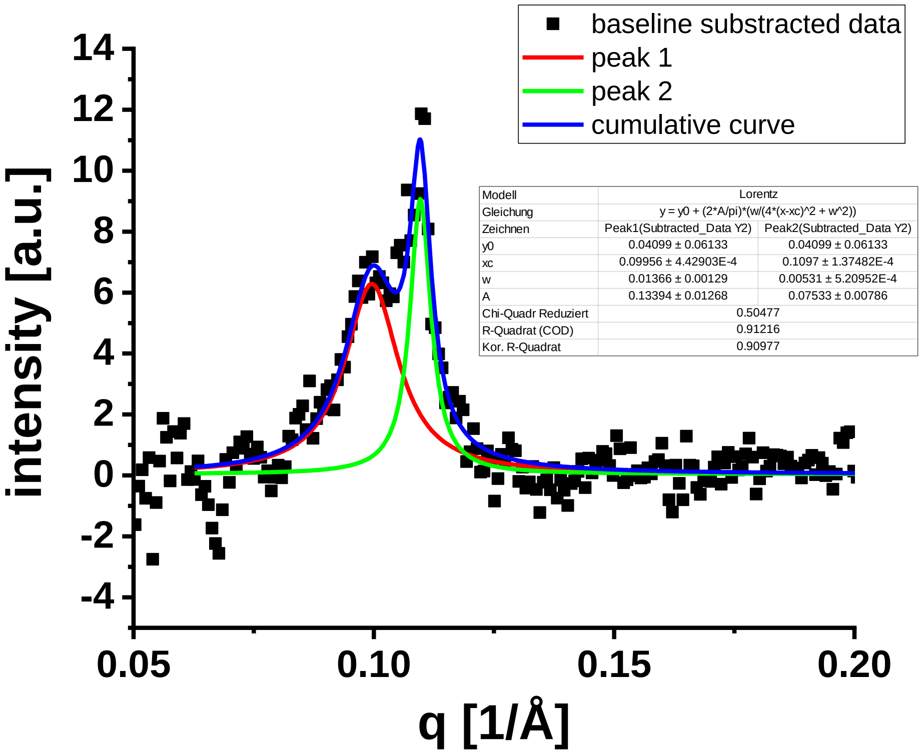

Figure S35: Multiple Lorentz fit of baseline subtracted GISAXS diffraction pattern of PEM-LPX-HA-CHI coating with the 2 x c(LPX) loading quantity.

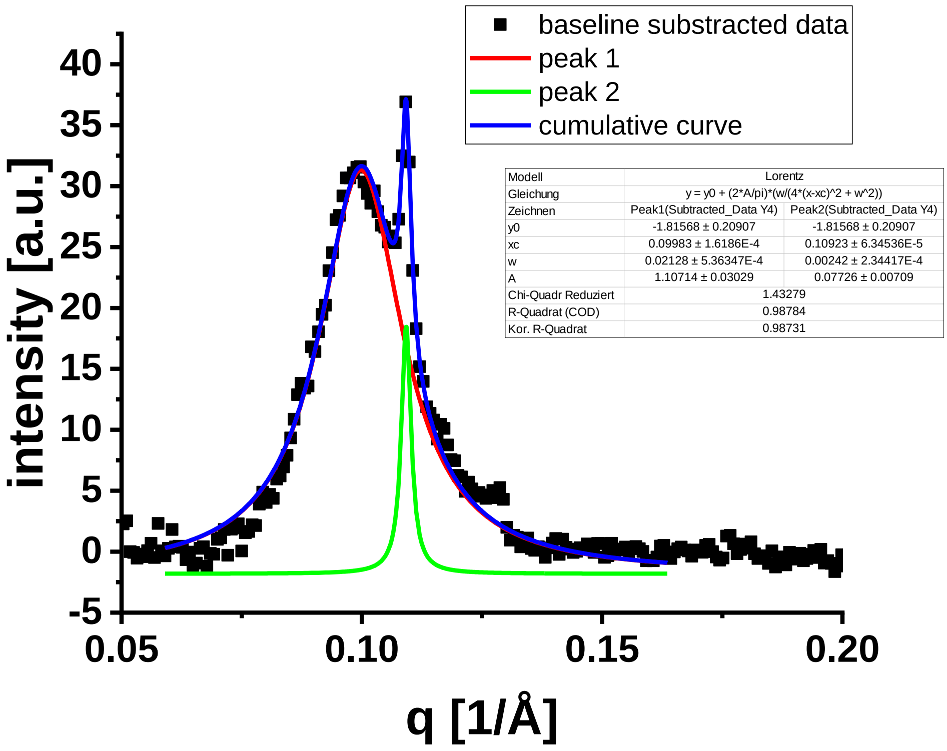

Figure S36: Multiple Lorentz fit of baseline subtracted GISAXS diffraction pattern of PEM-LPX-HA-CHI coating with the 4 x c(LPX) loading quantity.

Table S4: Peak intensities given as area of the Lorentz functions fitted to the Bragg-signal of PEMs with different LPX loading concentrations (see Figure 4 main text). The areas of the two fitted Lorentz functions are given (L1 and L2 area). Additionally, the ratios of area peak 1/area peak 2 (L1/L2 ratio) are calculated for the single positions and are also given as mean ± standard deviation (SD).

sample name	scan position	Lorentz peak fitting		area ratio L1/L2	
		L1 area ± error [a.u.]	L2 area ± error [a.u.]	single positions	mean ± SD
1 x c(LPX)	1	0.08 ± 0.009	0.04 ± 0.003	2	2.5 ± 1.2
	2	0.03 ± 0.006	0.03 ± 0.004	1	
	3	0.21 ± 0.014	0.04 ± 0.005	5.3	
	4	0.09 ± 0.017	0.05 ± 0.006	1.8	
2 x c(LPX)	1	0.03 ± 0.015	0.09 ± 0.010	0.3	1,5 ± 0.9
	2	0.13 ± 0.013	0.08 ± 0.008	1.6	
	3	0.24 ± 0.015	0.10 ± 0.012	2,4	
	4	0.22 ± 0.015	0.14 ± 0.011	1.6	
4 x c(LPX)	1	0.99 ± 0.036	0.07 ± 0.007	14	25 ± 17
	2	1.48 ± 0.042	0.03 ± 0.008	49	
	3	1.13 ± 0.041	0.05 ± 0.007	23	
	4	1.11 ± 0.030	0.08 ± 0.007	14	

Table S5: Area of the q_1 peaks (the mean of Σ (area peak 1, area peak 2) of Table S5) and the PEM bound DNA amount.

**sample name**	**area [a.u.]**	**PEM bound DNA amount [g/cm²]**
1 x c(LPX)	0.14 ± 0.08	0.69 ± 0.07
2 x c(LPX)	0.26 ± 0.11<br><br>*(1.9 * 1 x c(LPX) value)*	1.92 ± 0.17<br><br>*(2.8 * 1 x c(LPX) value)*
4 x c(LPX)	1.23 ± 0.20<br><br>*(8.8 * 1 x c(LPX) value)*	4.35 ± 0.12<br><br>*(6.30* 1 x c(LPX) value)*

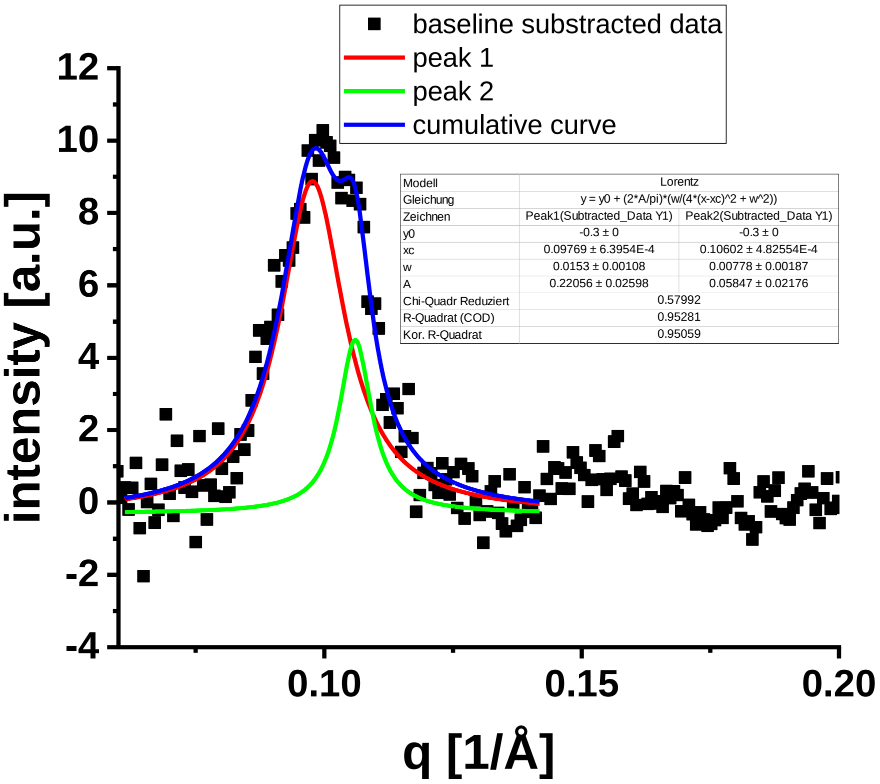

Figure S37: Multiple Lorentz fit of baseline subtracted GISAXS diffraction pattern of the in situ deposition experiment at time point 45 min.

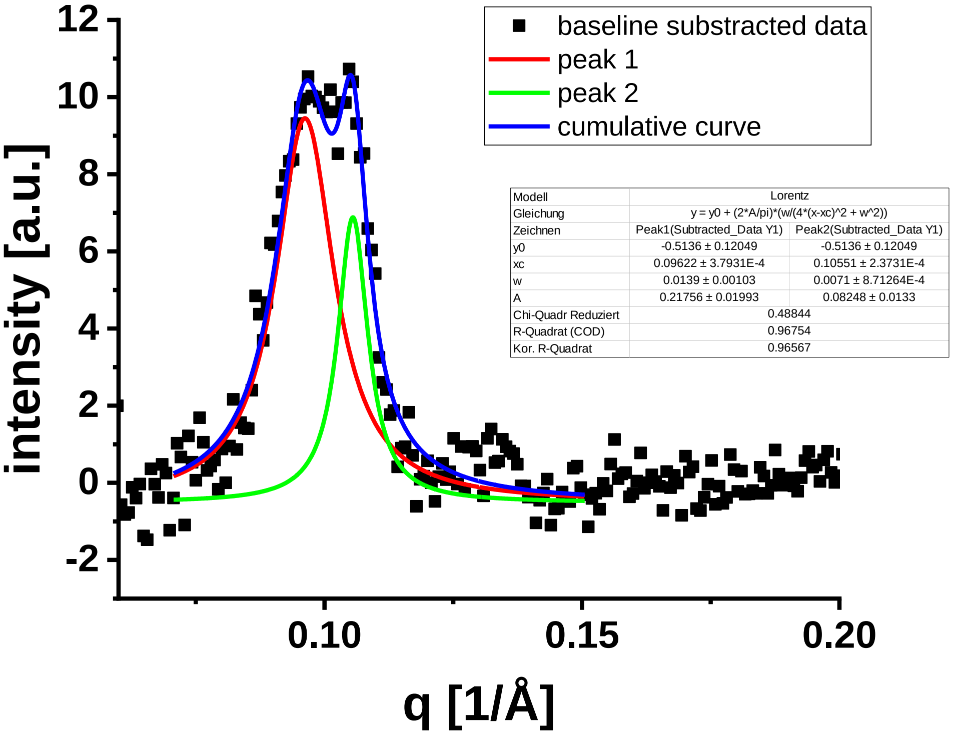

Figure S37: Multiple Lorentz fit of baseline subtracted GISAXS diffraction pattern of the in situ deposition experiment at time point 60 min.

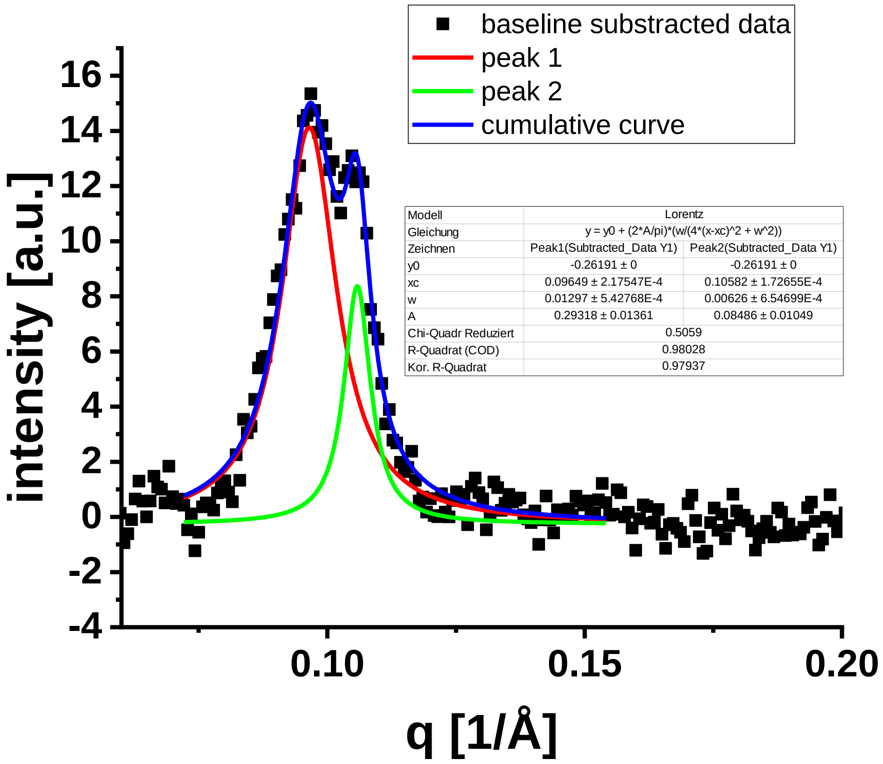

Figure S38: Multiple Lorentz fit of baseline subtracted GISAXS diffraction pattern of the in situ deposition experiment at time point 90 min.

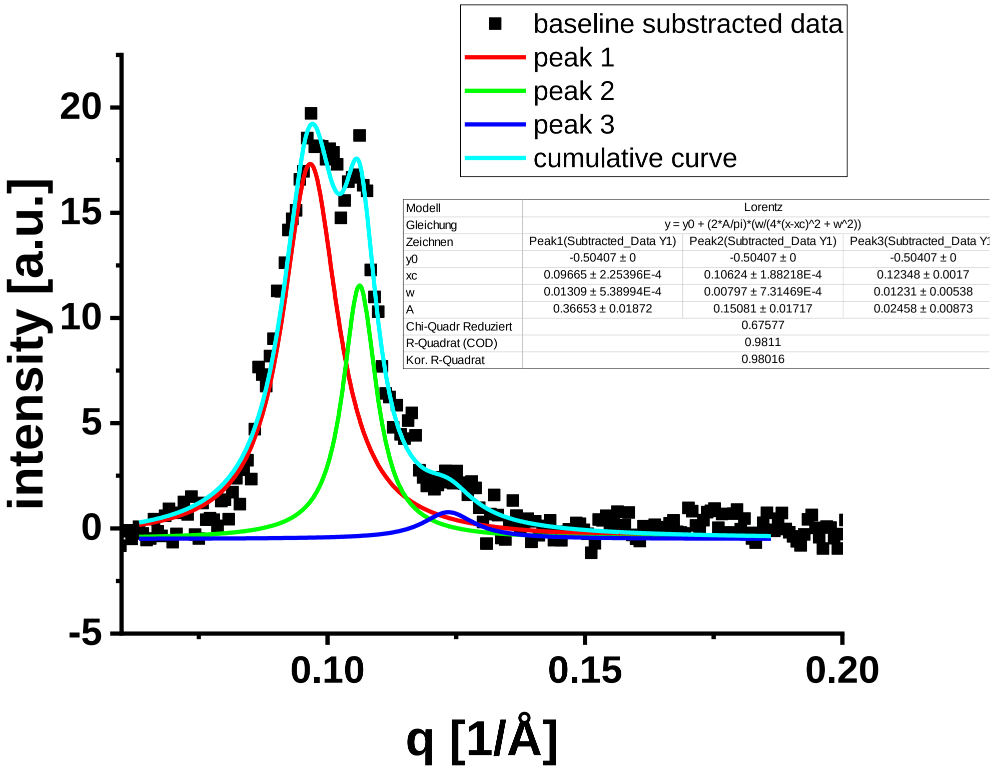

Figure S39: Multiple Lorentz fit of baseline subtracted GISAXS diffraction pattern of the in situ deposition experiment at time point 120 min.

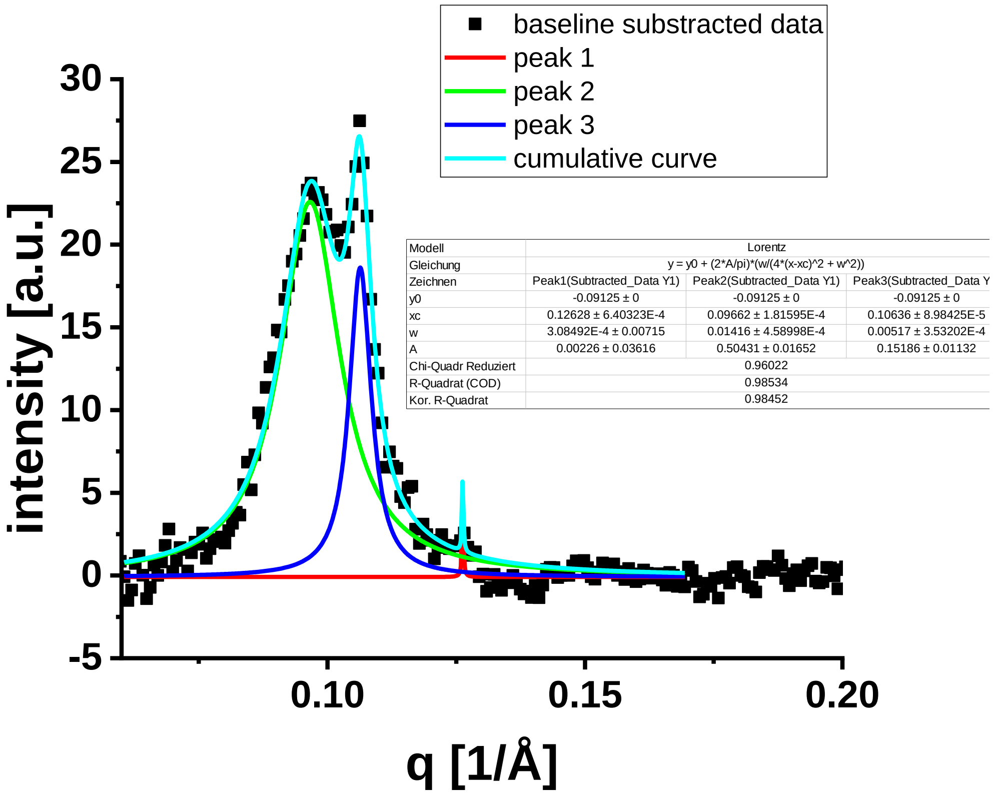

Figure S40: Multiple Lorentz fit of baseline subtracted GISAXS diffraction pattern of the in situ deposition experiment at time point 180 min.

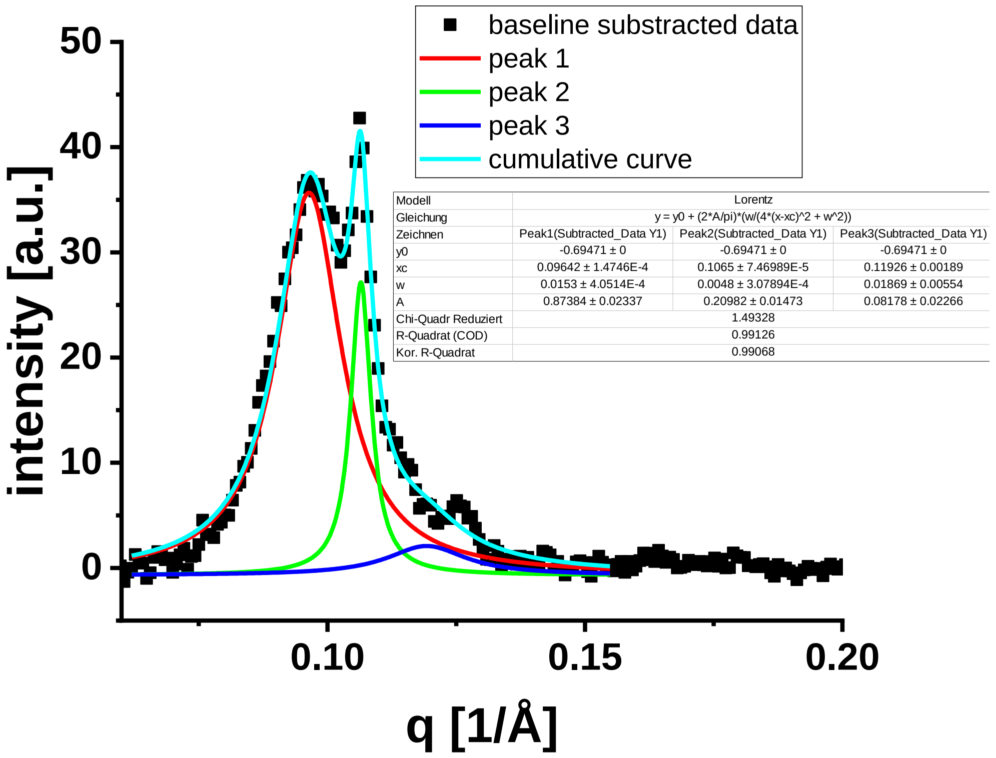

Figure S41: Multiple Lorentz fit of baseline subtracted GISAXS diffraction pattern of the in situ deposition experiment at time point 240 min.

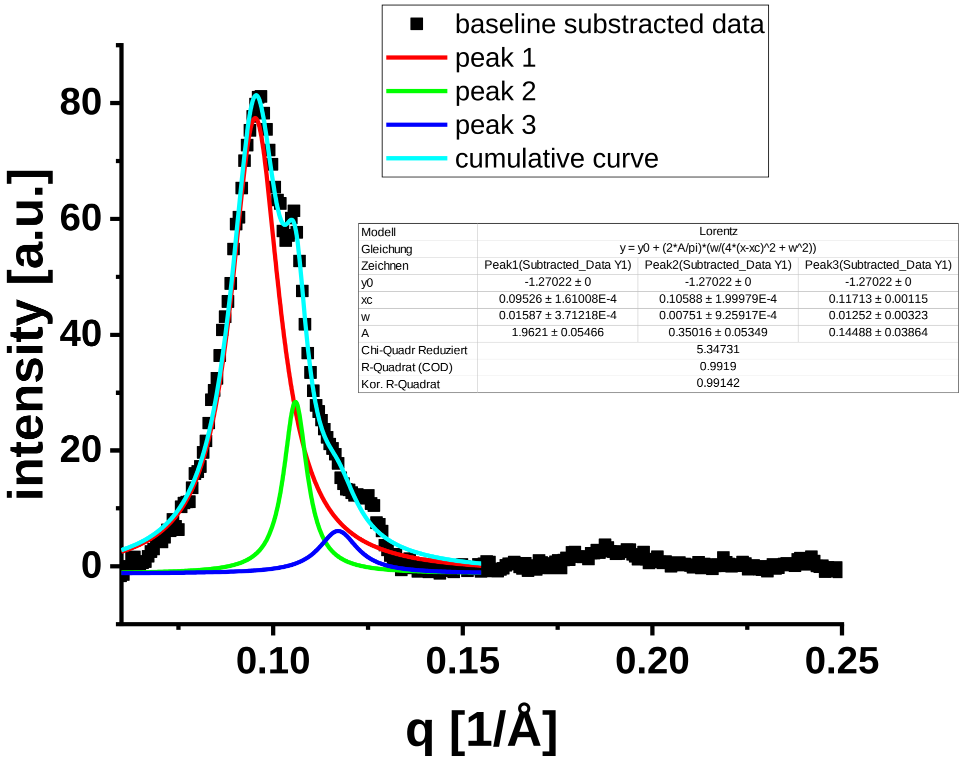

Modell	Lorentz		
Gleichung	y = y0 + (2*A/pi)*(w/(4*(x-xc)^2 + w^2))		
Zeichnen	Peak1(Subtracted_Data Y1)	Peak2(Subtracted_Data Y1)	Peak3(Subtracted_Data Y1)
y0	-1.27022 ± 0	-1.27022 ± 0	-1.27022 ± 0
xc	0.09526 ± 1.61008E-4	0.10588 ± 1.99979E-4	0.11713 ± 0.00115
w	0.01587 ± 3.71218E-4	0.00751 ± 9.25917E-4	0.01252 ± 0.00323
A	1.9621 ± 0.05466	0.35016 ± 0.05349	0.14488 ± 0.03864
Chi-Quadr Reduziert	5.34731		
R-Quadrat (COD)	0.9919		
Kor. R-Quadrat	0.99142		

Figure S42: Multiple Lorentz fit of baseline subtracted GISAXS diffraction pattern of the in situ deposition experiment at time point 540 min.

8. XRR measurement

The XRR data comparing the base PEM (HA, CHI)$_{25}$HA with the LPX-loaded PEM of the sequence (HA, CHI)$_{25}$HA-LPX-HA-CHI show no pronounced difference between both curves (Figure 4G). The curves show no clear Kiessig fringe or Bragg reflections. This can be caused by a low contrast in electron density of the components or by the absence of any ordering in the direction perpendicular to the surface. Further it has to be noted, that the thickness of the swollen PEM layer is larger than a µm, so that the fringes associated with the whole layer will not be resolved by the XRR experiment. The small feature observed for the PEM sample at ≈1.7 1/Å may be caused by the silicon oxide layer of the silicon wafer. According to earlier reports, HA/CHI multilayers are characterized by an exponential growth of loosely associated polyelectrolyte layers, by soft and hydrated structures, and the tendency of CHI to diffuse in the PEM (Kujawa et al., 2005) (Richert et al., 2004) (Almodóvar et al., 2011).

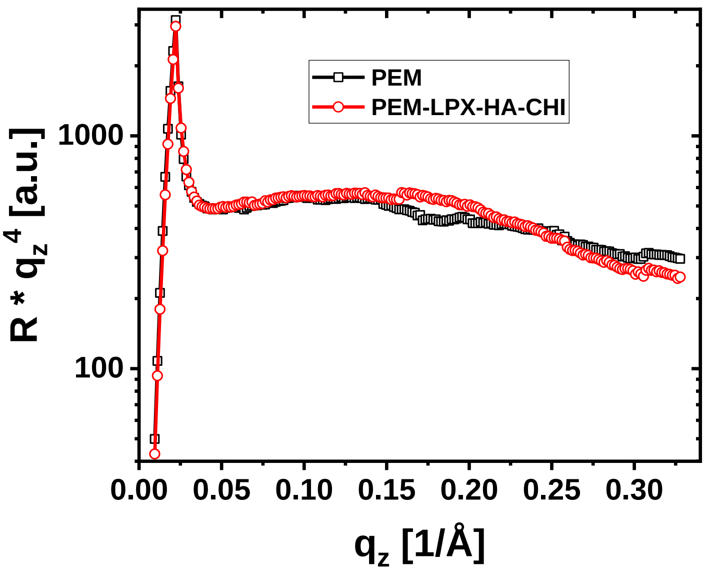

Figure S43: Reflectivity experiments of samples of the base PEM (HA, CHI)$_{25}$HA and the LPX loaded PEM of the sequence (HA, CHI)$_{25}$HA-LPX-HA-CHI in buffer.

References:

(1) Kujawa, P.; Moraille, P.; Sanchez, J.; Badia, A.; Winnik, F. M. Effect of Molecular Weight on the Exponential Growth and Morphology of Hyaluronan/Chitosan Multilayers: A Surface Plasmon Resonance Spectroscopy and Atomic Force Microscopy Investigation. *J. Am. Chem. Soc.* **2005**, *127* (25), 9224–9234. https://doi.org/10.1021/ja044385n.

(2) Richert, L.; Lavalle, P.; Payan, E.; Shu, X. Z.; Prestwich, G. D.; Stoltz, J.-F.; Schaaf, P.; Voegel, J.-C.; Picart, C. Layer by Layer Buildup of Polysaccharide Films: Physical Chemistry and Cellular Adhesion Aspects. *Langmuir* **2004**, *20* (2), 448–458. https://doi.org/10.1021/la035415n.

(3) Almodóvar, J.; Place, L. W.; Gogolski, J.; Erickson, K.; Kipper, M. J. Layer-by-Layer Assembly of Polysaccharide-Based Polyelectrolyte Multilayers: A Spectroscopic Study of Hydrophilicity, Composition, and Ion Pairing. *Biomacromolecules* **2011**, *12* (7), 2755–2765. https://doi.org/10.1021/bm200519y.
